# The KDM3A–KLF2–IRF4 axis maintains myeloma cell survival

**DOI:** 10.1038/ncomms10258

**Published:** 2016-01-05

**Authors:** Hiroto Ohguchi, Teru Hideshima, Manoj K. Bhasin, Gullu T. Gorgun, Loredana Santo, Michele Cea, Mehmet K. Samur, Naoya Mimura, Rikio Suzuki, Yu-Tzu Tai, Ruben D. Carrasco, Noopur Raje, Paul G. Richardson, Nikhil C. Munshi, Hideo Harigae, Takaomi Sanda, Juro Sakai, Kenneth C. Anderson

**Affiliations:** 1 Department of Medical Oncology, Dana-Farber Cancer Institute, Boston, Massachusetts 02215, USA; 2 BIDMC Genomics, Proteomics, Bioinformatics and Systems Biology Center, Beth Israel Deaconess Medical Center, Boston, Massachusetts 02115, USA; 3 MGH Cancer Center, Massachusetts General Hospital, Boston, Massachusetts 02114, USA; 4 Department of Biostatistics and Computational Biology, Dana-Farber Cancer Institute, Boston, Massachusetts 02215, USA; 5 West Roxbury Division, VA Boston Healthcare System, West Roxbury, MA 02132, USA; 6 Department of Hematology and Rheumatology, Tohoku University Graduate School of Medicine, Sendai, Miyagi 980-8574, Japan; 7 Cancer Science Institute of Singapore, Department of Medicine, National University of Singapore, Singapore 117599, Singapore; 8 Division of Metabolic Medicine, Research Center for Advanced Science and Technology, University of Tokyo, Tokyo 153-8904, Japan

## Abstract

KDM3A is implicated in tumorigenesis; however, its biological role in multiple myeloma (MM) has not been elucidated. Here we identify KDM3A–KLF2–IRF4 axis dependence in MM. Knockdown of *KDM3A* is toxic to MM cells *in vitro* and *in vivo*. KDM3A maintains expression of *KLF2* and *IRF4* through H3K9 demethylation, and knockdown of *KLF2* triggers apoptosis. Moreover, KLF2 directly activates *IRF4* and IRF4 reciprocally upregulates *KLF2*, forming a positive autoregulatory circuit. The interaction of MM cells with bone marrow milieu mediates survival of MM cells. Importantly, silencing of *KDM3A*, *KLF2* or *IRF4* both decreases MM cell adhesion to bone marrow stromal cells and reduces MM cell homing to the bone marrow, in association with decreased *ITGB7* expression in *MAF*-translocated MM cell lines. Our results indicate that the KDM3A–KLF2–IRF4 pathway plays an essential role in MM cell survival and homing to the bone marrow, and therefore represents a therapeutic target.

## Abstract (web summary)

Several histone modifiers have been implicated in the survival of multiple myeloma cells. Here, the authors reveal a role for the histone demethylase KDM3A in the survival of this haematologic cancer, and show that mechanistically KDM3A removes H3K9 methylation from the promoters of *KLF2* and *IRF4*, genes essential for myeloma cell survival.

## Graphical abstract

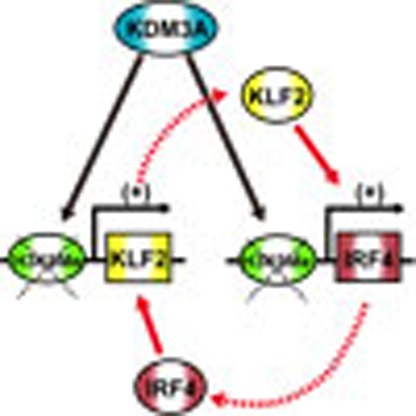

Histone methylation contributes to the regulation of chromatin structure and function, thereby modulating gene expression, including transcription factors. Thus, histone methylation dynamics are critical for numerous biological processes including cell cycle regulation, DNA damage and stress response, as well as development and differentiation^1^,^2^. Histone methylation is tightly regulated by methyltransferases and demethylases that mediate the addition and removal of this modification, and, importantly, dysregulation of histone methylation is implicated in the pathogenesis of cancers, including multiple myeloma (MM)^3^. For example, the t(4;14) (p16;q32) is present in 15–20% of MM patients, resulting in overexpression of *WHSC1* (also known as *MMSET*/*NSD2*), a histone H3 lysine 36 (H3K36) methyltransferase. Depletion of *WHSC1* expression induces apoptosis in MM cells with t(4;14) translocation^4^, whereas catalytically active WHSC1 promotes oncogenic transformation in an H3K36 dimethylation-dependent manner^5^. In addition, ∼10% of MM patients without the t(4;14) translocation have inactivating somatic mutations in *KDM6A* (also known as *UTX*), an H3K27 demethylase^6^. Thus, the role of histone methylation modifiers in myelomagenesis is of particular interest.

KDM3A (also known as JMJD1A), a member of the Jumonji C-domain-containing histone demethylases, catalyses removal of H3K9 mono- and dimethylation (H3K9me1 and H3K9me2)^7^ and functions as a coactivator for androgen receptor. It is also a crucial regulator of spermatogenesis, embryonic stem cell self-renewal, metabolic gene expression and sex determination^7^,^8^,^9^,^10^,^11^,^12^. Moreover, recent studies have implicated KDM3A in cancers^13^,^14^,^15^,^16^,^17^. However, little is currently known about the biological role of KDM3A in the pathogenesis of MM.

KLF2, a nuclear DNA-binding transcription factor of the Krüppel zinc-finger family, is involved in various biological processes including blood vessel and lung formation, as well as T-cell homeostasis^18^. KLF2 is also expressed in B cells^19^,^20^, and absence of KLF2 in B-cell compartment causes abnormalities in B-cell subset identity, cellular trafficking and humoral immunity^21^,^22^,^23^,^24^. Thus, KLF2 plays a crucial role in maintaining homeostasis of B cells and plasma cells; however, the functional significance of KLF2 in MM remains to be defined.

IRF4, a member of the interferon regulatory family of transcription factors, is highly expressed in B cells and plasma cells, and has an essential role in controlling B cell to plasma cell differentiation and immunoglobulin class switching^25^,^26^,^27^. Furthermore, IRF4 is involved in myelomagenesis. Specifically, a subset of MM harbours t(6;14)(p25;q32), which results in overexpression of *IRF4* (ref. 28). Moreover, not only this subset with translocation of *IRF4* but also all other subtypes of MM are dependent on IRF4 (ref. 29).

Here we investigate the biological significance of KDM3A in MM pathogenesis. We show that knockdown of *KDM3A* leads to apoptosis in MM cells, and that KDM3A directly upregulates *KLF2* and *IRF4* expression by removing H3K9 methyl marks at their promoters. We further show that knockdown of *KLF2* induces apoptosis, and that KLF2 directly transactivates *IRF4* promoter. Interestingly, *KLF2* is also a direct target of IRF4, forming a positive autoregulatory loop in MM cells. In addition, we demonstrate that silencing of *KDM3A*, *KLF2* or *IRF4* impairs MM cell homing to the bone marrow. These findings suggest that the KDM3A–KLF2–IRF4 axis plays an essential role in MM cell growth and homing to the bone marrow, and therefore represents a potential therapeutic target.

## Results

### KDM3A is indispensable for MM cell survival

**Figure 1:**
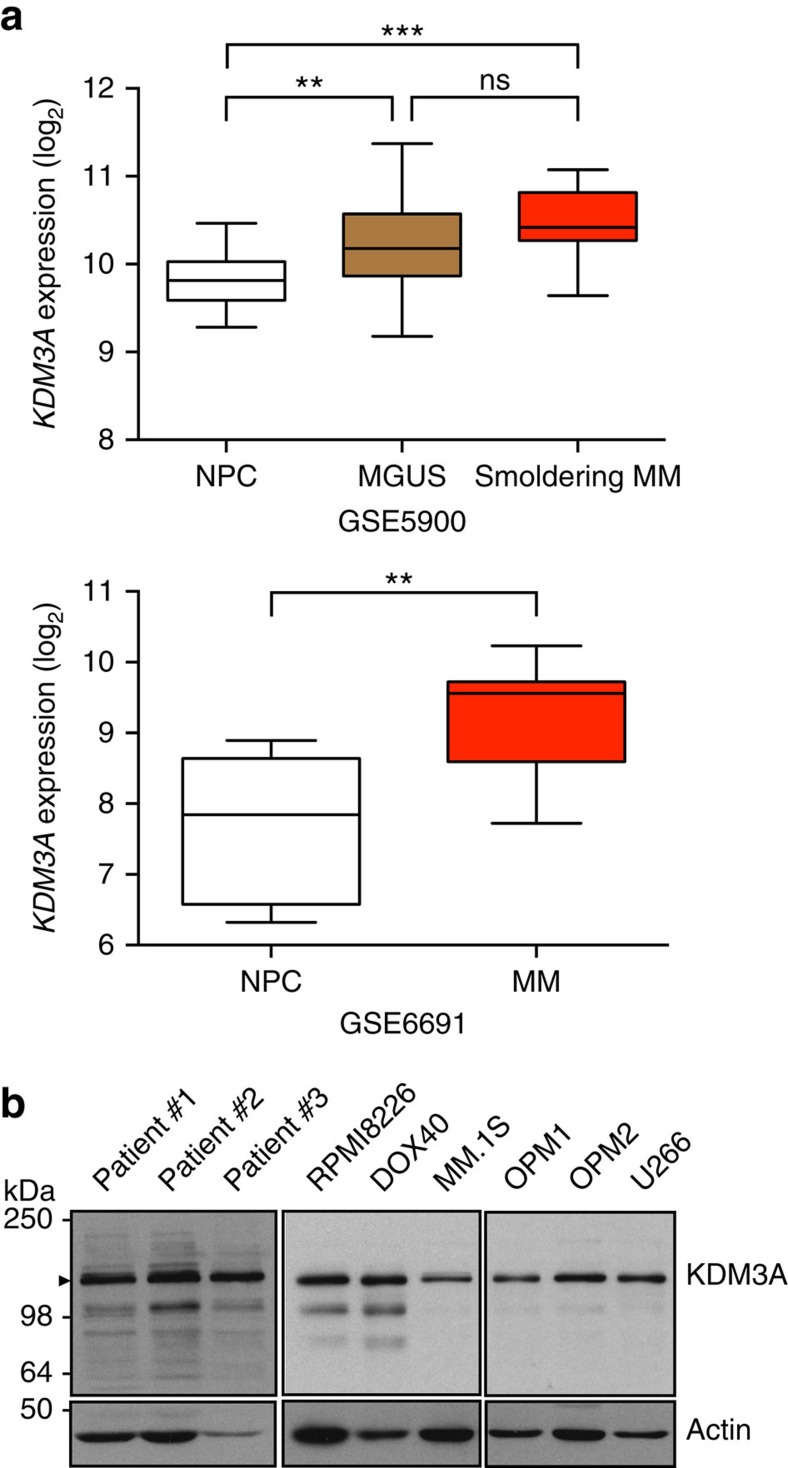
KDM3A expression in MM cells. (**a**) *KDM3A* mRNA expression in patient MM samples. Publicly available microarray data sets (GSE5900 and GSE6691) were analysed for mRNA expression of *KDM3A* in normal plasma cells (NPC), monoclonal gammopathy of undetermined significance (MGUS), smoldering multiple myeloma (Smoldering MM) and MM cells. **P<0.01, ***P<0.001. ns, not significant; analysis of variance (ANOVA) followed by Tukey's test. (**b**) Immunoblot analysis of KDM3A in patient MM cells and MM cell lines. Arrowhead represents KDM3A. Data are representative of at least two independent experiments, except for patient samples' blot.

**Figure 2:**
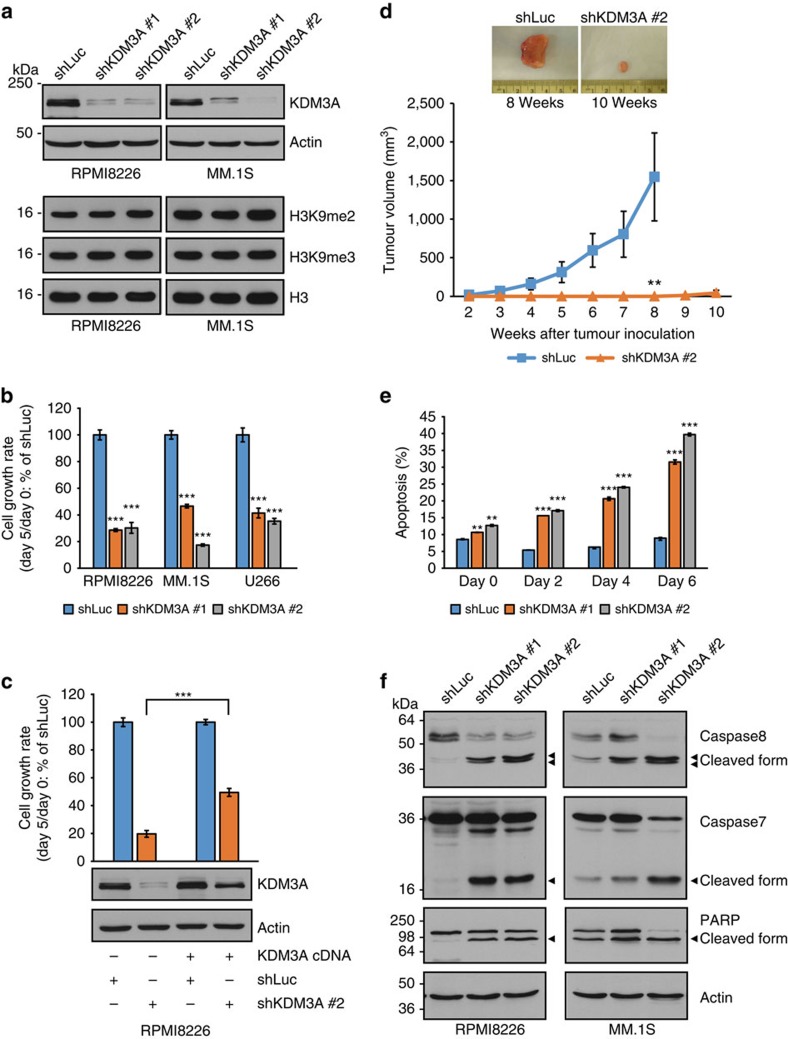
KDM3A is indispensable for the survival of MM cells. (**a**) Immunoblot analysis of KDM3A, H3K9me2 and H3K9me3 in *KDM3A*-knockdown RPMI8226 and MM.1S cells. Cells were transduced with either *KDM3A*-specific shRNA (shKDM3A #1 and #2) or shLuc by lentivirus. Whole-cell lysates were subjected to immunoblot analysis. Actin or H3 served as the loading control for each membrane. (**b**) MM cell lines (RPMI8226, MM.1S and U266) were transduced with shKDM3A or control shLuc. Three days post infection, which was designated as day 0, cells were plated in 96-well plates. Cell viability was measured on days 0 and 5 by MTT assay, and the cell growth rate (day 5/day 0) relative to shLuc was determined. (**c**) RPMI8226 cells were retrovirally transduced with the *KDM3A* cDNA carrying synonymous mutations in the shKDM3A #2 target sequence or with empty vector. Cells stably expressing the *KDM3A* cDNA or empty vector were then lentivirally transduced with shKDM3A #2 or shLuc. The cell growth rate (day 5/day 0) after lentiviral infection was determined for shKDM3A relative to shLuc. The growth rate for control shLuc in each cell type expressing the *KDM3A* cDNA or empty vector is set as 100%. (**d**) MM.1S cells transduced with shKDM3A #2 or shLuc (4 × 10^6^ viable cells) were subcutaneously injected into SCID mice. Data represent mean±s.e.m. *n*=7 mice per group. An image of a representative tumour in each group is shown (top panel). (**e**) Apo2.7 immunostaining in RPMI8226 cells after knockdown of *KDM3A*. Cells were analysed 3 days post infection (day 0) and on days 2, 4, 6 for apoptosis by measuring apo2.7-positive cells. (**f**) Immunoblot analysis of caspase 8, caspase 7 and PARP in whole-cell lysates from *KDM3A*-knockdown RPMI8226 and MM.1S cells. Arrowheads indicate cleaved form. Actin served as the loading control. For **b**,**c**,**e**, data represent mean±s.d. of quintuplicate cultures (**b**,**c**) or duplicate cultures (**e**). For **a**–**c**,**e**,**f**, data are representative of at least two independent experiments. **P<0.01, ***P<0.001 compared with control; Student's *t*-test.

We first evaluated expression of *KDM3A* mRNA in MM patient samples using publicly available gene expression profiling data because this jumonji demethylase has been implicated in the pathogenesis of several other cancers^13^,^14^,^15^,^16^,^17^. In two independent data sets^30^,^31^, *KDM3A* expression was significantly elevated in monoclonal gammopathy of undetermined significance and MM patient samples compared with normal plasma cells (Fig. 1a). We next examined KDM3A protein expression in MM cells. KDM3A protein was detected by immunoblotting in three patient MM cells and six human MM cell lines tested (Fig. 1b). This signal was increased by overexpression of *KDM3A* (Supplementary Fig. 1) and decreased by silencing of *KDM3A* (Fig. 2a), confirming specific detection of KDM3A protein. Hence, we hypothesized that KDM3A may also play a role in the pathogenesis of MM.

To evaluate the functional role of KDM3A, MM cell lines were transduced with short hairpin RNAs (shRNAs) targeting *KDM3A* (shKDM3A #1 and #2) or control shRNA targeting *luciferase* (shLuc) by lentivirus. Transduction of *KDM3A*-specific shRNAs reduced mRNA and protein levels of KDM3A in RPMI8226 and MM.1S cells (Fig. 2a and Supplementary Fig. 2a). Although KDM3A is a demethylase specific to H3K9me1 and me2 (ref. 7), the global H3K9me2 level was not significantly increased in MM cells transduced with *KDM3A*-specific shRNAs, similar to short interfering RNA-mediated *KDM3A* knockdown in HeLa cells^32^ (Fig. 2a). Importantly, knockdown of *KDM3A* significantly inhibited MM cell growth (Fig. 2b and Supplementary Fig. 2b), which was partially rescued by expression of the *KDM3A* cDNA carrying silent mutations in the shKDM3A-targeting sequence (Fig. 2c). Consistent with cell growth inhibition, DNA synthesis was also significantly reduced in MM cells transduced with shRNA targeting *KDM3A* versus control shRNA (Supplementary Fig. 2c). To further assess the effect of *KDM3A* knockdown on MM cell growth *in vivo*, we subcutaneously injected MM.1S cells transduced with shRNA against *KDM3A* or shLuc into severe combined immunodeficient (SCID) mice. As shown in Fig. 2d, cell growth was significantly reduced in shKDM3A-treated MM.1S cells compared with shLuc-treated cells.

We next examined the molecular mechanism of cell growth inhibition. Quantitative analysis of apoptosis with flow cytometry using apo2.7 staining showed that apoptotic cells were significantly increased in *KDM3A*-knockdown RPMI8226 cells (Fig. 2e). Furthermore, immunoblot analysis revealed increased cleavage of caspase-8, -7 and poly (ADP-ribose) polymerase (PARP) after knockdown of *KDM3A* in RPMI8226, MM.1S and U266 cells (Fig. 2f and Supplementary Fig. 2d). Silencing of *KDM3A* had little effect on the cell cycle profile (Supplementary Fig. 2e). These results suggest that knockdown of *KDM3A* triggers MM cell cytotoxicity via apoptosis.

### KDM3A activates *KLF2* and *IRF4* through H3K9 demethylation

**Figure 3:**
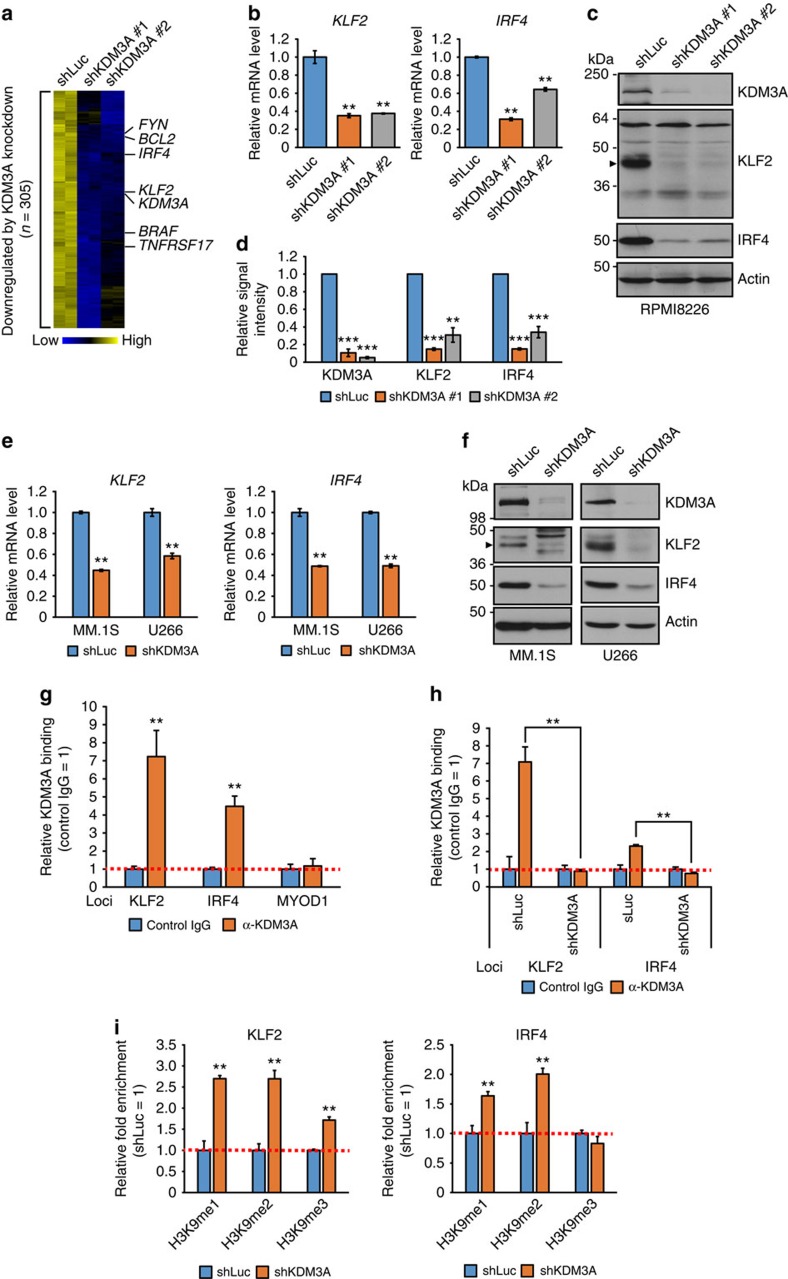
KDM3A directly regulates *KLF2* and *IRF4* expression through H3K9 demethylation at their promoters in MM cells. (**a**) Heatmap depicting the relative gene expression in RPMI8226 cells transduced with two independent shRNAs targeting *KDM3A* (shKDM3A #1 and shKDM3A #2) or shLuc. A total of 305 probes were selected based on ≥1.5-fold downregulation in *KDM3A*-knockdown cells and clustered. (**b**–**d**) Quantitative real-time PCR (**b**) and immunoblot (**c**,**d**) analysis of KLF2 and IRF4 in RPMI8226 cells transduced with either shKDM3A or shLuc. After 3 days of infection, which is defined as day 0 in Fig. 2, cells were harvested for isolation of total RNA or whole-cell lysates. (**b**) Values represent the amount of mRNA relative to shLuc, defined as 1. (**c**) Arrowhead represents KLF2. (**d**) Signal intensity of each immunoblot was quantified using the ImageJ software. Results were normalized by Actin and are shown as relative signal intensity (shLuc=1). Error bars represent s.e.m of three independent experiments. (**e**,**f**) Quantitative real-time PCR (**e**) and immunoblot (**f**) analysis of KLF2 and IRF4 in MM.1S and U266 cells transduced with either shKDM3A or shLuc. After 3 days of infection, cells were harvested for isolation of total RNA or whole-cell lysates. (**g**) ChIP analysis showing KDM3A occupancy on *KLF2* and *IRF4* core promoters in RPMI8226 cells. Results were normalized to control IgG. The *MYOD1* promoter region was used as negative control. (**h**) KDM3A occupancy is abrogated by *KDM3A* knockdown on *KLF2* and *IRF4* promoter regions in RPMI8226 cells. RPMI8226 cells transduced with either shKDM3A or shLuc were used for ChIP, followed by quantitative real-time PCR. (**i**) Enrichment of H3K9 methylation by *KDM3A* knockdown on *KLF2* and *IRF4* promoter regions in RPMI8226 cells. ChIP assays were performed with RPMI8226 cells transduced with either shKDM3A or shLuc. The relative enrichment over the input was assessed. Results are shown as fold enrichment compared with control shLuc. For **b**,**e**,**g**–**i**, error bars represent s.d. of triplicate measurements. For **b**–**i**, data are representative of at least two independent experiments. **P<0.01, ***P<0.001 compared with control; Student's *t*-test.

To identify the downstream effector targets of KDM3A, we next examined gene expression profiles after knockdown of *KDM3A* in RPMI8226 cells. With a cutoff of ≥1.5-fold downregulation in *KDM3A*-knockdown cells relative to control cells, a total of 305 probe sets were downregulated in *KDM3A*-knockdown cells (Fig. 3a and Supplementary Data 1). The list includes genes encoding anti-apoptotic protein BCL2 (ref. 33), essential receptor for plasma cell survival TNFRSF17 (also known as BCMA)^34^,^35^, *BRAF*^36^,^37^ and *FYN*^38^ (Fig. 3a). Among these putative KDM3A targets, a gene of particular interest is *KLF2*, a key molecule in maintenance of B cell and plasma cell phenotype and function^21^,^22^,^23^,^24^. Another relevant gene is *IRF4*, given its known crucial role in MM cell survival^29^.

To validate the microarray results, we measured *KLF2* and *IRF4* mRNA using quantitative real-time PCR (QRT–PCR) and confirmed that expression of *KLF2* and *IRF4* was significantly reduced in *KDM3A*-silenced RPMI8226 cells (Fig. 3b). Immunoblot analysis further confirmed that protein levels of KLF2 and IRF4 were consistently diminished after knockdown of *KDM3A* (Fig. 3c,d). We identified a band at ∼40 kDa as KLF2 protein, since this signal was increased by overexpression of *KLF2* (Supplementary Fig. 3a, right panel) and decreased by silencing of *KLF2* (Supplementary Fig. 3b, right panel), reflecting mRNA levels (Supplementary Fig. 3a,b, left panels). Knockdown of *KDM3A* also decreased *KLF2* and *IRF4* expression at both the mRNA and protein levels in MM.1S and U266 MM cell lines (Fig. 3e,f).

To determine whether KDM3A directly regulates *KLF2* and *IRF4* expression, we next analysed KDM3A binding to *KLF2* and *IRF4* core promoters (near the transcriptional start sites) using chromatin immunoprecipitation (ChIP) assay in RPMI8226 cells. As a negative control we used the *MYOD1* promoter region, since this cell line had no *MYOD1* expression, and silencing of *KDM3A* had no effect on its expression. As shown in Fig. 3g, KDM3A bound to the promoters of *KLF2* and *IRF4*, but not to the *MYOD1* promoter. Moreover, knockdown of *KDM3A* abrogated KDM3A binding to *KLF2* and *IRF4* promoters, indicating that these are specific signals (Fig. 3h). We next evaluated H3K9 methylation levels on *KLF2* and *IRF4* promoters after silencing of *KDM3A*. H3K9me1 and me2 levels were significantly increased at both promoter regions in *KDM3A*-knockdown cells compared with control cells (Fig. 3i). Interestingly, we observed an increased trimethyl-H3K9 (H3K9me3) level at the *KLF2* promoter, as was the case at *Prm1* and *Tnp1* promoters in the spermatids of *KDM3A*-deficient mice^8^, although KDM3A is unable to demethylate H3K9me3 *in vitro*^7^. Taken together, these findings suggest that KDM3A directly regulates *KLF2* and *IRF4* expression by demethylating H3K9 methyl marks at their promoters in MM cells.

### KLF2 is required for MM cell survival

**Figure 4:**
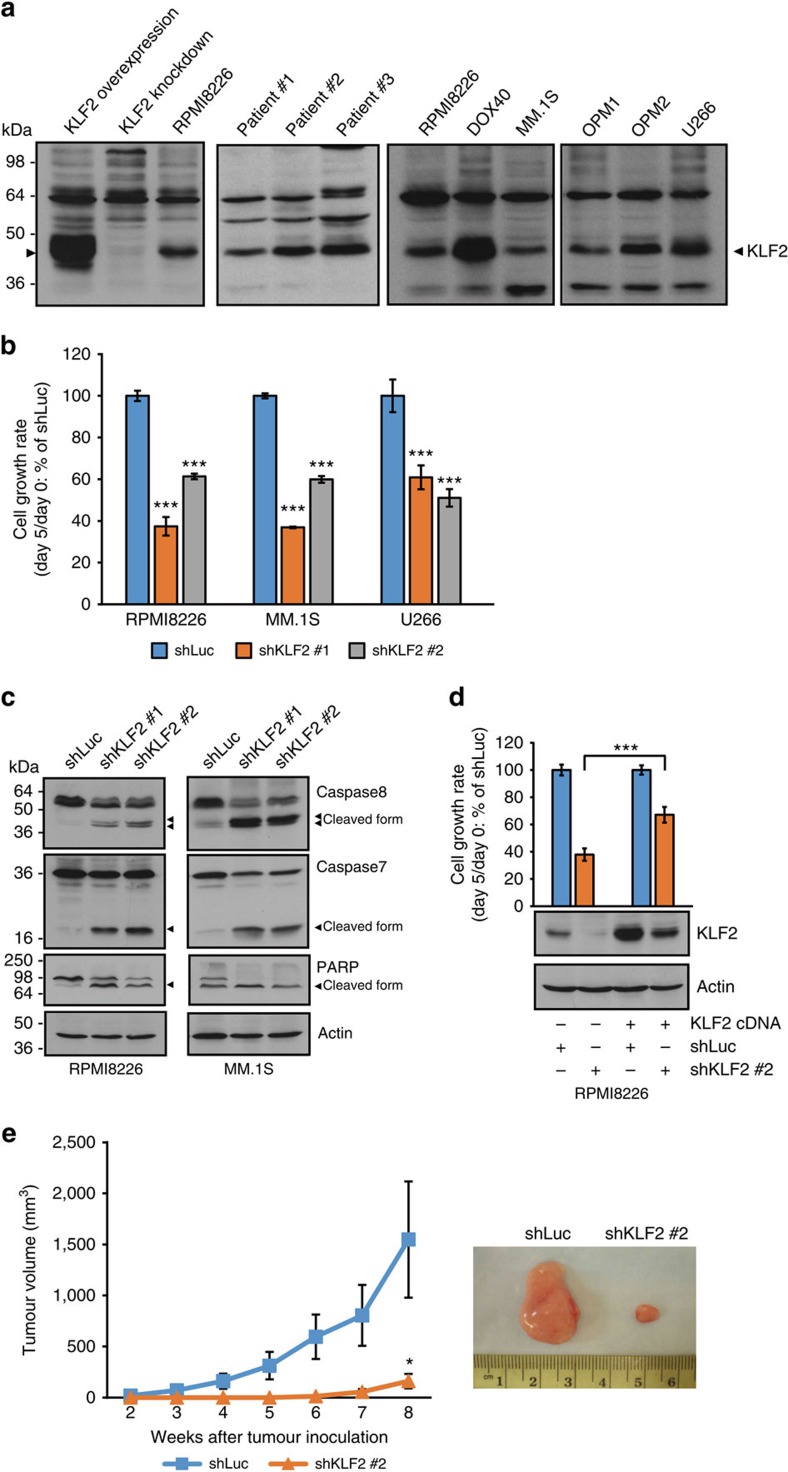
KLF2 is required for MM cell survival. (**a**) Immunoblot analysis of KLF2 in patient MM cells and MM cell lines. Arrowhead represents KLF2. Whole-cell lysates from *KLF2*-overexpressed or knockdown RPMI8226 cells were used as positive or negative control for KLF2 antibody (N2212), respectively. (**b**) MM cell lines (RPMI8226, MM.1S and U266) were transduced with either *KLF2*-specific shRNA (shKLF2 #1 and #2) or control shLuc by lentivirus. Cell viability was measured on days 0 and 5 by MTT assay, and the cell growth rate (day 5/day 0) relative to shLuc was determined. (**c**) Immunoblot analysis of caspase 8, caspase 7 and PARP in *KLF2*-knockdown RPMI8226 and MM.1S cells. Arrowheads indicate cleaved form. Actin served as the loading control. (**d**) RPMI8226 cells expressing the *KLF2* cDNA carrying synonymous mutations in the shKLF2 #2 target sequence or empty vector were transduced with shKLF2 #2 or shLuc by lentivirus. The growth rate for control shLuc in each cell type expressing the *KLF2* cDNA or empty vector is set as 100%. (**e**) MM.1S cells transduced with shKLF2 #2 or shLuc (4 × 10^6^ viable cells) were subcutaneously injected into SCID mice. Data represent mean±s.e.m. *n*=7 mice per group. An image of a representative tumour in each group is shown (right panel). Data of control shLuc group are the same data shown in Fig. 2d since subcutaneous xenograft studies of MM.1S cells treated with shKDM3A and shKLF2 were performed simultaneously with a common control shLuc group. For **b**,**d**, data represent mean±s.d. of quintuplicate cultures. For **a**–**d**, data are representative of at least two independent experiments, except for patient samples' blot (**a**). *P<0.05, ***P<0.001 compared with control; Student's *t*-test.

We next confirmed that KLF2 protein was highly expressed in patient MM cells and MM cell lines (Fig. 4a). To investigate the biological significance of KLF2 in MM cells, shRNAs targeting *KLF2* (shKLF2 #1 and #2) or shLuc were transduced into MM cells by lentivirus. We confirmed that the *KLF2* expression level was decreased after knockdown of *KLF2* using QRT–PCR and immunoblotting (Supplementary Fig. 3b). Importantly, silencing of *KLF2* significantly reduced cell growth of MM cell lines (Fig. 4b and Supplementary Fig. 4a), inhibited DNA synthesis (Supplementary Fig. 4b) as well as triggered apoptosis, evidenced by caspase-8, -7 and PARP cleavage (Fig. 4c). Expression of *KLF2* cDNA carrying synonymous mutations in the shKLF2-targeting sequence partially rescued RPMI8226 cells from growth inhibition induced by shKLF2 (Fig. 4d). Significantly decreased *in vivo* growth of MM.1S cells transduced with shRNA against *KLF2* compared with shLuc-transduced cells was noted in a subcutaneous xenograft model in SCID mice (Fig. 4e). Collectively, these data support the notion that KLF2 sustains MM cell survival.

### *IRF4* is a direct transcriptional target of KLF2 in MM cells

**Figure 5:**
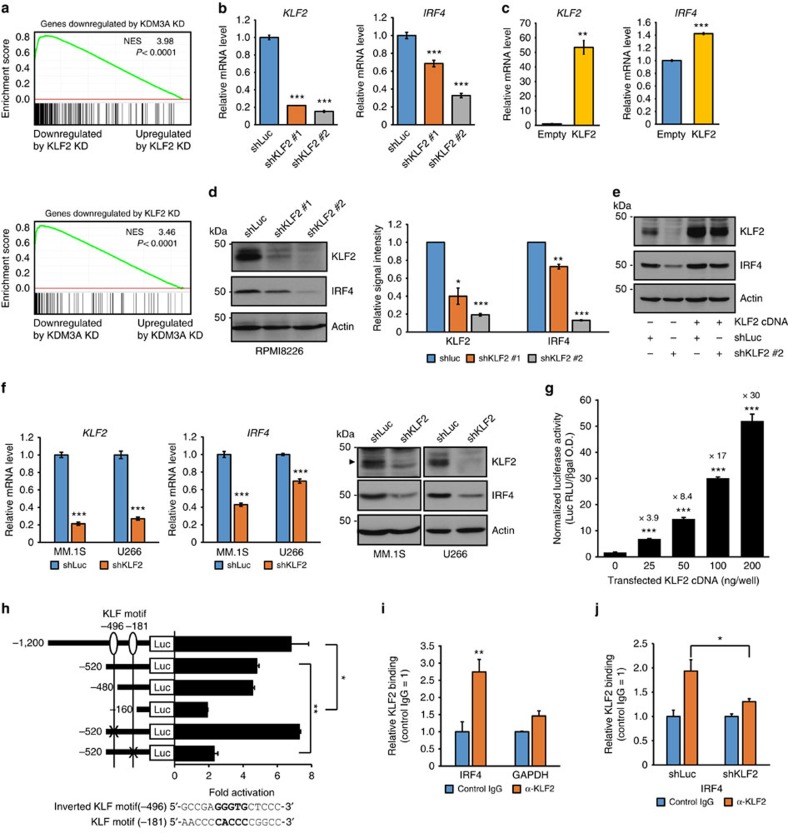
*IRF4* is a direct transcriptional target of KLF2 in MM cells. (**a**) Microarray analysis in RPMI8226 cells transduced with shKDM3A, shKLF2 or shLuc was performed. The genes significantly downregulated by *KDM3A* or *KLF2* knockdown compared with control were used as gene sets for the GSEA. Normalized enrichment score (NES) and *P* values are shown. (**b**,**c**) Quantitative real-time PCR of *KLF2* and *IRF4* after knockdown (**b**) and overexpression (**c**) of *KLF2* in RPMI8226 cells. (**d**) Immunoblot analysis of KLF2 and IRF4 after knockdown of *KLF2* in RPMI8226 cells. Shown are the relative signal intensity (shLuc=1) normalized by Actin. Error bars represent s.d. of two independent experiments. (**e**) RPMI8226 cells expressing the *KLF2* cDNA carrying synonymous mutations in the shKLF2 #2 target sequence or empty vector were transduced with shKLF2 #2 or shLuc. Whole-cell lysates were subjected to immunoblot analysis. (**f**) Quantitative real-time PCR and immunoblot analysis of KLF2 and IRF4 after knockdown of *KLF2* in MM.1S and U266 cells. (**g**) Transactivation of the *IRF4* promoter by KLF2. The indicated amounts of *KLF2* expression plasmids were transfected into 293T cells together with the human *IRF4* promoter-luciferase reporter. The value above each bar indicates the induction level compared with empty vector. (**h**) 293T cells were co-transfected with the indicated *IRF4*-luciferase reporter and 0.04 μg of *KLF2* expression plasmid or empty vector, and then assayed for luciferase activity. The fold activation (normalized luciferase activity co-transfected with *KLF2* expression plasmid relative to empty vector) is shown. (**i**) ChIP analysis showing KLF2 occupancy on *IRF4* promoter in RPMI8226 cells. *GAPDH* promoter was used as negative control. (**j**) KLF2 occupancy is abrogated by *KLF2* knockdown on *IRF4* promoter in RPMI8226 cells. RPMI8226 cells transduced with either shKLF2 or shLuc were used for ChIP. For **b**,**c**,**f**,**i**,**j**, error bars represent s.d. of triplicate measurements. For **g**,**h**, data represent mean±s.d. of three (**g**) or two (**h**) biological replicates. For **b**–**j**, data are representative of at least two independent experiments. *P<0.05, **P<0.01, ***P<0.001 compared with control; Student's *t*-test.

To identify the downstream targets of KLF2 in MM cells, we carried out microarray analysis after knockdown of *KLF2* in RPMI8226 cells (Supplementary Data 2). Gene set enrichment analysis (GSEA) showed significant correlation between the genes downregulated by silencing of *KDM3A* and *KLF2*, indicating that KLF2 is a major downstream effector of KDM3A in MM cells (Fig. 5a). Interestingly, we found that a gene encoding IRF4 was also significantly decreased after knockdown of *KLF2* (Supplementary Data 2). To validate the *KLF2* microarray data, we used QRT–PCR to show that *IRF4* mRNA was significantly decreased in *KLF2*-silenced RPMI8226 cells (Fig. 5b). In a complementary manner, expression of *IRF4* was increased in *KLF2*-overexpressed RPMI8226 cells (Fig. 5c). Consistent with the mRNA level, IRF4 protein expression was reduced after knockdown of *KLF2* (Fig. 5d), and expression of KLF2 protein rescued IRF4 protein expression after knockdown of *KLF2* (Fig. 5e). Depletion of *KLF2* also resulted in decreased *IRF4* expression at the mRNA and protein levels in MM.1S and U266 cells (Fig. 5f).

To identify the mechanisms regulating *IRF4* expression, we next performed promoter reporter assays in 293T cells using reporter constructs containing a DNA fragment of the human *IRF4* promoter (between bp −1,200 and +14). As shown in Fig. 5g, expression of KLF2 significantly increased luciferase expression in a dose-dependent manner, suggesting that KLF2 transactivates the *IRF4* promoter. There are two KLF consensus-binding motifs within 1.2-kb region of the *IRF4* promoter (Fig. 5h). To examine whether both elements were required to mediate effects of KLF2, we introduced a series of 5′ deletions or mutations of KLF motifs into the promoter region. Deletion of the sequence from −1,200 to −480 had minimal effects on the response to ectopic expression of KLF2 (Fig. 5h). A further deletion to −160 almost abolished KLF2-mediated promoter activation. Furthermore, mutation of the proximal KLF motif significantly reduced the transactivation by KLF2, whereas mutation of the distal KLF motif did not affect the sensitivity to KLF2 expression. These results indicate that this proximal KLF site is at least one of the pivotal sites for transactivation of the *IRF4* gene by KLF2. There remained weak KLF2-mediated promoter activation in the construct that has a deletion or a mutation of this proximal KLF motif. Although the *IRF4* promoter within a 160-bp region does not contain KLF consensus-binding motif (5′-CACCC-3′), this region includes GC-rich sites. Sp1-like/KLF proteins have affinities for different GC-rich sites^18^; therefore, there may be another potential KLF2-binding site in this proximal promoter.

Finally, to assess KLF2 binding to the *IRF4* promoter in MM cells, we carried out ChIP assays in RPMI8226 cells using antibody against KLF2 or control IgG, followed by QRT–PCR. As shown in Fig. 5i, endogenous KLF2 bound to the *IRF4* proximal promoter. This ChIP signal was significantly reduced after knockdown of *KLF2*, indicating that this signal is KLF2-specific (Fig. 5j). Taken together, these data provide evidence that *IRF4* is a direct target of KLF2 in MM cells.

### *KLF2* is a direct target of IRF4 transactivation in MM cells

**Figure 6:**
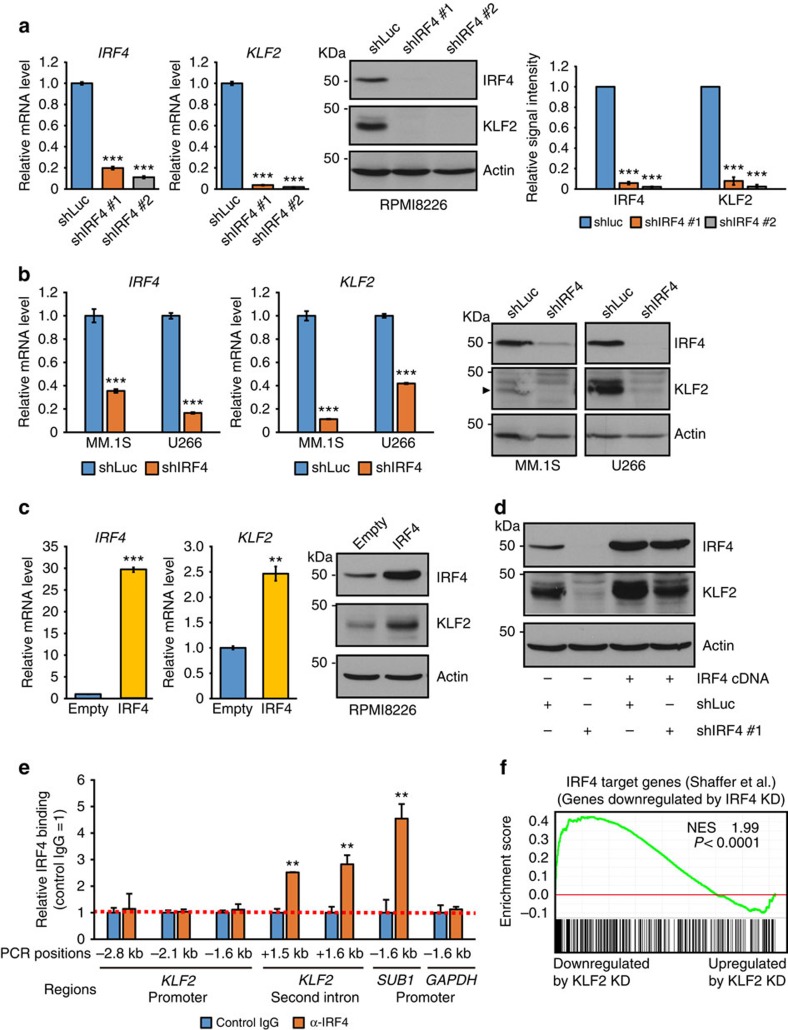
*KLF2* is a direct target of IRF4 transactivation in MM cells. (**a**,**b**) Quantitative real-time PCR and immunoblot analysis of IRF4 and KLF2 in RPMI8226 (**a**), MM.1S and U266 (**b**) cells transduced with either shRNA targeting *IRF4* (shIRF4) or shLuc. (**a**, right panel) Shown are the relative signal intensity (shLuc=1) normalized by Actin. Error bars represent s.e.m. of three independent experiments. (**c**) Quantitative real-time PCR and immunoblot analysis of IRF4 and KLF2 after overexpression of *IRF4* in RPMI8226 cells. (**d**) RPMI8226 cells expressing the *IRF4* cDNA or empty vector were transduced with shIRF4 #1 that targets the 3′ untranslated region of IRF4 or shLuc by lentivirus. Whole-cell lysates were subjected to immunoblot analysis with antibodies against IRF4 and KLF2. (**e**) ChIP analysis showing IRF4 occupancy on *KLF2* second intron in RPMI8226 cells. ChIP assays were performed for the regions that contain tandem IRF4 motifs on *KLF2* promoter and intron (indicated PCR positions are relative to the transcriptional start site). Results were normalized to control IgG. The *SUB1* promoter^29^ or *GAPDH* promoter was used as positive or negative control, respectively. (**f**) Significant correlation between the genes downregulated by *KLF2* knockdown and *IRF4* knockdown in MM cells. The genes significantly downregulated by *IRF4* knockdown^29^ were used as gene set for GSEA. NES and *P* values are shown. For **a** (left panel), **b**,**c**,**e**, error bars represent s.d. of triplicate measurements. For **a**–**e**, data are representative of at least two independent experiments. **P<0.01, ***P<0.001 compared with control; Student's *t*-test.

Since transcription factors have been reported to form an autoregulatory feedback loop to sustain their expression in some models^29^,^39^,^40^, we hypothesized that IRF4 might regulate *KLF2* expression. As expected, knockdown of *IRF4* downregulated *KLF2* expression at both the mRNA and protein levels in three MM cell lines (Fig. 6a,b). Furthermore, overexpression of *IRF4* upregulated *KLF2* expression (Fig. 6c), and expression of IRF4 protein partially rescued KLF2 protein expression after knockdown of *IRF4* in RPMI8226 cells (Fig. 6d). We next ascertained whether IRF4 directly binds to the *KLF2* locus. ChIP assays demonstrated that IRF4 bound to *KLF2* second intron that contains tandem IRF4 motifs (GAAA), but not to the regions that harbour tandem IRF4 motifs, on the *KLF2* promoter in RPMI8226 cells (Fig. 6e). These data indicate that *KLF2* is a direct target of IRF4 in MM cells.

Collectively, these results suggest that KLF2 activates *IRF4* expression and *vice versa*, forming a positive autoregulatory loop in MM cells. Consistent with these findings, the set of genes downregulated by *IRF4* knockdown^29^ was significantly enriched for genes downregulated by *KLF2* knockdown (Fig. 6f).

### The KDM3A KLF2 IRF4 axis sustains MM cell adhesion and homing

**Figure 7:**
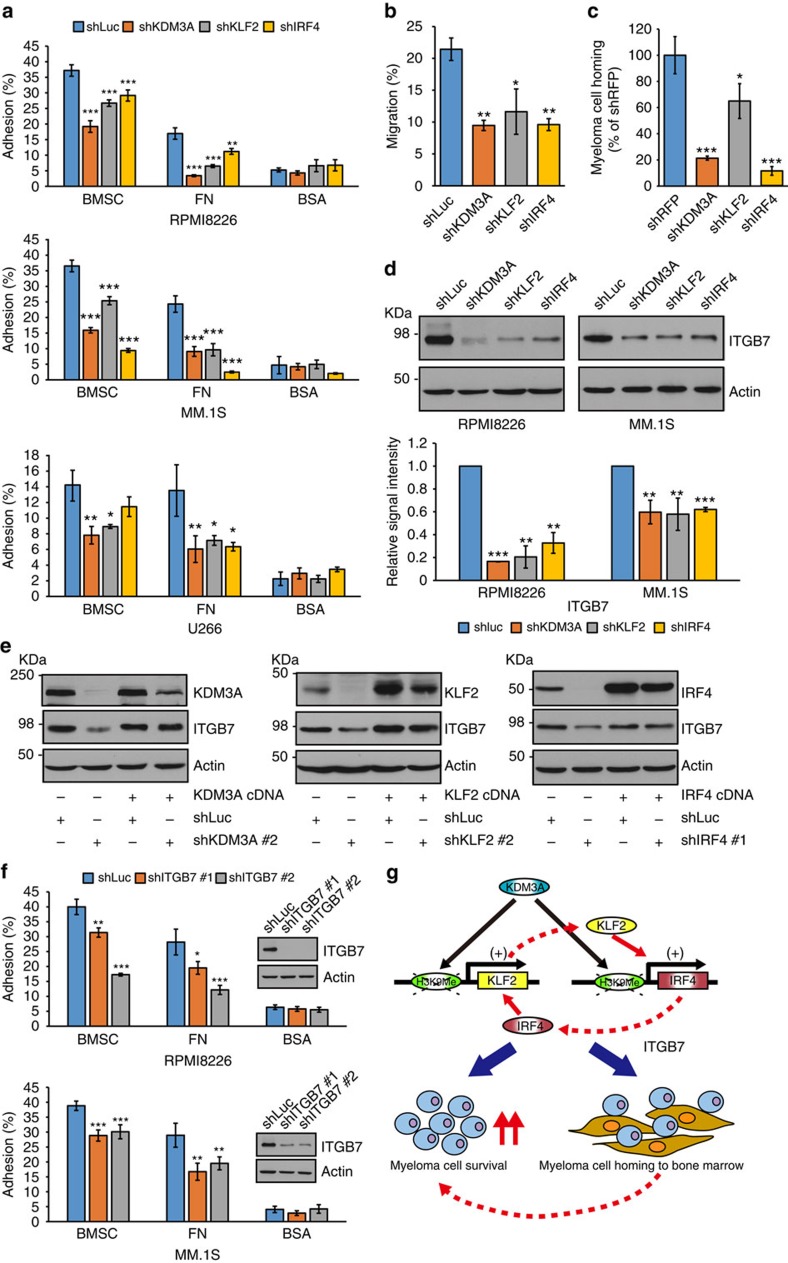
The KDM3A–KLF2–IRF4 axis contributes to adhesion and homing of MM cells to the bone marrow. (**a**) RPMI8226, MM.1S and U266 cells expressing each shRNA were labelled with calcein-AM and incubated in bone marrow stromal cells (BMSC)- or fibronectin (FN)-coated plates. After washing plates, adherent cells were quantified using a fluorescence plate reader. Results are shown as per cent adhesion over the input. BSA-coated wells were used as negative control. (**b**) MM.1S cells transduced with each shRNA were placed in the upper chamber of a modified Boyden chamber and incubated with CXCL12 (20 nM) added to the lower chamber. After 4 h, cells that migrated to the lower chamber were quantified. Results are shown as per cent migration over the input. (**c**) MM.1S-Luc-GFP cells expressing each shRNA (15 × 10^6^ viable cells) were intravenously injected into NOD-SCID mice. Twenty-four hours after injection, bone marrow cells of recipient mice were collected, and GFP-positive cells were detected using flow cytometry. Results are shown as homing efficacy relative to shRFP cells. Data represent mean±s.e.m. *n*=4–5 mice per group. *P<0.05, ***P<0.001 compared with control; ANOVA followed by Dunnett's test. (**d**) Immunoblot analysis of ITGB7 after knockdown of *KDM3A*, *KLF2*, *IRF4* in RPMI8226 and MM.1S cells. Shown are the relative signal intensity (shLuc=1) normalized by Actin. Error bars represent s.d. of two (RPMI8226) or three (MM.1S) independent experiments. (**e**) RPMI8226 cells expressing each cDNA were transduced with each shRNA as shown. Whole-cell lysates were subjected to immunoblot analysis. Data are representative of two independent experiments. (**f**) Adhesion assays were performed with RPMI8226 and MM.1S cells treated with either shITGB7 or shLuc. (**g**) Model of the KDM3A–KLF2–IRF4 axis in MM cells. KDM3A demethylates H3K9 of *KLF2* and *IRF4* promoter, resulting in upregulation of *KLF2* and *IRF4*. KLF2 and IRF4 mutually activate expression of each other. The KDM3A–KLF2–IRF4 axis maintains MM cell survival and homing to the bone marrow. For **a**,**b**,**f**, data represent mean±s.d. of quadruplicate incubations (**a**,**f**), or triplicate incubations (**b**). *P<0.05, **P<0.01, ***P<0.001 compared with control; Student's *t*-test.

The bone marrow microenvironment plays a crucial role in the pathogenesis of MM^41^,^42^. In this context, KLF2 has been reported to control homing of plasma cells to the bone marrow^24^. We therefore hypothesized that the KDM3A–KLF2–IRF4 axis might regulate adhesion and homing of MM cells to the bone marrow. To test this notion, we first evaluated the effect of silencing of *KDM3A*, *KLF2* or *IRF4* on MM cell adhesion and migration *in vitro*. As shown in Fig. 7a, knockdown of *KDM3A*, *KLF2* or *IRF4* decreased adhesion of RPMI8226, MM.1S and U266 cells to bone marrow stromal cells and fibronectin, without affecting the adhesion to bovine serum albumin (BSA), which served as a negative control. Knockdown of *KDM3A*, *KLF2* or *IRF4* also reduced MM.1S migratory ability towards CXCL12 in a transwell migration assay (Fig. 7b). We finally investigated the effect of silencing of *KDM3A*, *KLF2* or *IRF4* on MM cell homing to the bone marrow *in vivo*. Quantitative analysis of the bone marrow homing of MM.1S-Luc-GFP cells was performed. An equal number of MM.1S-Luc-GFP cells stably expressing shRNA targeting *KDM3A*, *KLF2* or *IRF4* was injected via tail vein into sublethally irradiated nonobese diabetic (NOD)-SCID mice. Bone marrow cells of femurs were harvested 24 h after cell injection, and green fluorescent protein (GFP)-positive cells were detected using flow cytometry. The bone marrow homing of *KDM3A*, *KLF2* or *IRF4*-depleted MM.1S cells was significantly reduced compared with control cells expressing shRNA against red fluorescent protein (*RFP*) (Fig. 7c).

Previous studies have shown that reduced plasma cell homing to the bone marrow is accompanied by decreased *ITGB7* expression in B-cell-specific *KLF2* knockout mice^24^, and that ITGB7 modulates MM cell adhesion and homing to the bone marrow^43^,^44^. We therefore next hypothesized that the KDM3A–KLF2–IRF4 axis might control expression of *ITGB7* in MM cells. Of note, silencing of *KDM3A*, *KLF2* or *IRF4* downregulated expression of ITGB7 in RPMI8226 and MM.1S, which are *MAF*-translocated cell lines with high ITGB7 expression (Fig. 7d and Supplementary Fig. 5a). This reduced ITGB7 expression was restored by expression of each of these cDNAs in RPMI8226 cells, indicating that decreased ITGB7 expression after knockdown of *KDM3A*, *KLF2* or *IRF4* is not an off-target effect (Fig. 7e). In contrast, knockdown of *KDM3A* or *KLF2* did not downregulate other MM cell-relevant adhesion molecules such as *ITGB1*, *ITGA4*, *ITGA5*, *MUC1*, *SDC1*, *CD44*, *CD147* and *ICAM1* (Supplementary Fig. 5b). We then evaluated MM cell adhesion after knockdown of *ITGB7*. Consistent with previous observations^43^,^44^, knockdown of *ITGB7* reduced adhesion of RPMI8226 and MM.1S cells to bone marrow stromal cells and fibronectin (Fig. 7f). Taken together, these results suggest that the KDM3A–KLF2–IRF4 axis regulates *ITGB7* expression in *MAF*-translocated MM cells, and regulates, at least in part, MM cell adhesion and homing to the bone marrow.

## Discussion

KDM3A has recently been implicated in the development of various solid tumours including colorectal cancer, sarcoma, bladder cancer and lung cancer^13^,^14^,^15^,^16^,^17^. The specific roles of KDM3A in the pathogenesis of cancers are now being delineated. For example, Cho *et al.*^17^ showed that KDM3A regulated the *HOXA1* gene, which in turn suppressed *CCND1* transcription, resulting in G1/S arrest in cancer cells. In the current study, we demonstrate that silencing of *KDM3A* induces apoptosis in MM cells. Unlike other cancers, knockdown of *KDM3A* does not affect *HOXA1* expression or cell cycle distribution in MM cells (Supplementary Fig. 2e). Rather our current data demonstrate that KDM3A activates expression of *KLF2* and *IRF4* in MM cells. Depletion of *IRF4* has been shown to induce MM cell death^29^. Our data also show that silencing of *KLF2* triggers growth inhibition, indicating that not only IRF4, but also KLF2, are effector molecules in the KDM3A-dependent survival pathway. This notion is supported by GSEA, which shows that the list of genes downregulated by *KDM3A* silencing is significantly enriched for genes downregulated by *KLF2* silencing. Nevertheless, overexpression of *KLF2* or *IRF4* did not rescue *KDM3A* knockdown-mediated cell growth inhibition (Supplementary Fig. 6a). In this study, we show that KDM3A sustains *KLF2* and *IRF4* expression through H3K9 demethylation at their promoters. Furthermore, KLF2 and IRF4 mutually transactivate expression of each other, generating a positive feedback loop in MM cells (Fig. 7g). These data suggest that not only each transcription factor but also KDM3A are indispensable for the expression of another transcription factor. In fact, overexpression of *KLF2* did not rescue IRF4 expression in *KDM3A*-knockdown cells as well as overexpression of *IRF4* did not rescue KLF2 expression (Supplementary Fig. 6b). These results suggest that overexpression of either *KLF2* or *IRF4* is not sufficient to rescue the *KDM3A*-knockdown phenotype, since both KLF2 and IRF4 are crucial in MM cell survival. KDM3A and these transcription factors may also cooperatively regulate the transcription machinery of common target genes critical for MM cell survival and therefore KLF2 or IRF4 may not be enough to rescue the effect of *KDM3A* depletion, although further studies are required to address this issue. Thus, our data support the view that KDM3A maintains MM cell survival by coordinately regulating expression of essential transcription factors through histone demethylation. An interesting question is whether KDM3A contributes to the pathogenesis of other haematologic malignancies by the same mechanism, given its high expression at the same level as MM (Supplementary Fig. 7a). Interestingly, knockdown of *KDM3A* also significantly impaired growth of diffuse large B-cell lymphoma (DLBCL) cell lines (Supplementary Fig. 7b). However, knockdown of *KDM3A* did not decrease *KLF2* and *IRF4* expression in DLBCL cell lines (Supplementary Fig. 7c), suggesting that KDM3A sustains survival of DLBCL via a different mechanism than that for MM. These data suggest a cancer-type-specific role of KDM3A.

The bone marrow microenvironment promotes MM cell survival^41^,^42^. Thus, adhesion and homing of MM cells to the bone marrow plays a key role in their growth and survival. Our study demonstrates that the KDM3A–KLF2–IRF4 pathway modulates MM cell adhesion and homing to the bone marrow, suggesting that this pathway maintains MM cell survival not only by preventing apoptosis but also by enhancing the interaction of MM cells with the bone marrow microenvironment (Fig. 7g). We found that knockdown of *KDM3A*, *KLF2* or *IRF4* downregulates *ITGB7* expression in *MAF*-translocated MM cells. ITGB7 mediates MAF-driven MM cell adhesion to bone marrow stromal cells^44^, as well as MM cell homing to the bone marrow^43^. ITGB7 may therefore contribute, at least in part, to the KDM3A–KLF2–IRF4 axis-dependent adhesion of MM cells to bone marrow stromal cells. Moreover, knockdown of *KDM3A*, *KLF2* or *IRF4* impairs adhesion of U266 MM cells, which only expressed low level of *ITGB7*, suggesting that this pathway mediates adhesion through not only ITGB7 but also via other unknown mechanisms.

Recent studies have described the genetic landscape of MM using whole-genome- and whole-exome sequencing, copy number array and cytogenetic analysis using samples from a large number of patients^36^,^45^,^46^. These studies identified mutated oncogenes including *KRAS*, *NRAS* and *BRAF*, as well as copy number gains of these oncogenes. One missense mutation in *KDM3A* was found, but it was not recurrent and its functional significance is unknown^45^. Moreover, no copy number alterations or chromosomal rearrangements involving the *KDM3A* locus were identified^45^,^46^. Nevertheless, MM cells are addicted to KDM3A. Thus, KDM3A addiction in myeloma may be ‘non-oncogene addiction,' a concept proposed by Luo *et al.*^47^ to describe the increased dependence of cancer cells on normal cellular activities of certain genes and pathways that are not inherently oncogenic by themselves. We found that *KDM3A* expression is elevated in patient MM cells relative to normal plasma cells (Fig. 1a). The MAF family of transcription factors, known driver oncogenes in MM^44^,^48^,^49^, may be associated with this aberrant *KDM3A* expression in a subset of MM, since *KDM3A* expression is modestly but significantly elevated in the subgroup of newly diagnosed MM patients with *MAF* translocations and high *MAF* expression (Supplementary Fig. 8a)^50^. The bone marrow microenvironment may also regulate *KDM3A* expression in MM cells with disease progression, since *KDM3A* transcript levels in MM cell lines were significantly upregulated after co-culture with bone marrow stromal cells (Supplementary Fig. 8b)^42^.

The present study demonstrates that knockdown of *KDM3A* induces apoptosis in MM cells. *KDM3A*-deficient mice exhibit spermatogenesis defects, male-to-female sex reversal and adult onset obesity, but no other additional phenotypes^8^,^10^,^11^,^12^. Hence, there may be a therapeutic window allowing for safely targeting KDM3A. A recent study revealed that KDM3A was distinct from other Jumonji C family members in both its structure and function. KDM3A forms a homodimer through its catalytic domains, and catalyses removal of H3K9 methylation via a two-step process in which two active sites of the dimer are crucial for the enzymatic activity^51^. This reaction mechanism is totally different from catalytic processes of other JumonjiC H3K9 demethylases such as PHF8 and KIAA1718, which neither form a homodimer nor catalyse successive two-step methylation^52^. These studies may provide the framework for developing small molecules that specifically inhibit KDM3A.

In conclusion, we identify KDM3A as a crucial epigenetic regulator of MM cell survival. We also show that KLF2 and IRF4 are downstream effectors in the KDM3A-dependent survival pathway, and that KLF2 and IRF4 regulate expression of each other. In addition, we demonstrate that this pathway also sustains MM cell adhesion and homing to the bone marrow, suggesting further supporting MM cell survival. Our data therefore suggest that modulation of histone H3K9 methylation by inhibiting KDM3A represents a promising novel therapeutic strategy in MM, and provide the preclinical rationale for development of potent selective drugs targeting KDM3A for clinical evaluation in MM.

## Methods

### Cell culture

Human MM cell lines, RPMI8226, MM.1S and U266B1 (U266), were purchased from American Type Culture Collection (ATCC), and the identity of these cell lines was recently confirmed using STR profiling (Promega GenePrint 10 System). 293T packing cell line was also obtained from ATCC. MM.1S cell line expressing luciferase and GFP, MM.1S-Luc-GFP, was a kind gift from Dr Irene Ghobrial (Dana-Farber Cancer Institute, Boston, USA). Doxorubicin-resistant RPMI-DOX40 (DOX40) cell line was kindly provided by Dr William Dalton (Lee Moffitt Cancer Center, Tampa, USA). Human plasma cell leukaemia cell lines, OPM1 and OPM2, were kindly provided by Dr Edward Thompson (University of Texas Medical Branch, Galveston, USA). Human DLBCL cell lines, HBL-1 and SU-DHL-4, were kindly provided by Drs Bjoern Chapuy and Margaret A. Shipp (Dana-Farber Cancer Institute). Human DLBCL cell line KIS-1 (refs 53, 54) was kindly provided by Dr Shinsuke Iida (Nagoya City University, Nagoya, Japan). All MM cell lines and KIS-1 were maintained in Roswell Park Memorial Institute (RPMI)-1640 medium containing 100 U ml^−1^ penicillin and 100 μg ml^−1^ streptomycin, supplemented with 10% (v/v) fetal bovine serum and 2 μM L-glutamine, in 5% CO_2_ at 37 °C. The growth media for SU-DHL-4 were further supplemented with 10 mM HEPES. The growth media for HBL-1 were further supplemented with 10 mM HEPES and 1 mM sodium pyruvate. 293T cells were maintained in Dulbecco's Modified Eagle's Medium (DMEM) containing 100 U ml^−1^ penicillin and 100 μg ml^−1^ streptomycin, supplemented with 10% (v/v) fetal bovine serum.

Cell lines were tested to rule out mycoplasma contamination using the MycoAlert Mycoplasma Detection Kit (Lonza).

### Primary MM cells and bone marrow stromal cells

Bone marrow samples were obtained from MM patients after informed consent and approval by the Institutional Review Board of the Dana-Farber Cancer Institute. Mononuclear cells were separated by Ficoll-Paque PLUS (GE Healthcare), and MM cells were purified by CD138-positive selection with anti-CD138 magnetic activated cell separation microbeads (Miltenyi Biotec). Long-term bone marrow stromal cells were established by culturing CD138-negative bone marrow mononuclear cells for 4–6 weeks in DMEM containing 100 U ml^−1^ penicillin and 100 μg ml^−1^ streptomycin, supplemented with 15% (v/v) fetal bovine serum^55^.

### Gene expression analysis using publicly available data sets

Gene Expression Omnibus data sets (accession numbers GSE5900 (ref. 30), GSE6691 (ref. 31), GSE36133 (ref. 56), GSE2658 (ref. 50) and GSE20540 (ref. 42)) were used for gene expression analysis. 212689_s_at is the probe for KDM3A transcript on Affymetrix Human Genome U133A Array or Human Genome U133 Plus 2.0 Array.

### Immunoblot analysis

Cells were harvested, washed with PBS and lysed in RIPA buffer (Boston Bioproducts) containing a mixture of protease inhibitor (cOmplete, Mini, Roche) and 1 mM phenylmethanesulfonyl fluoride (Sigma-Aldrich). The suspension was incubated for 30 min at 4 °C and centrifuged at top speed in a microfuge for 15 min at 4 °C. The supernatant was used as whole-cell lysates. For immunoblotting of histone protein, cells were lysed in TOPEX buffer (300 mM NaCl, 50 mM Tris-HCl, pH7.5, 1% SDS, 0.5% Triton X-100, 1 mM dithiothreitol and 33.33 unit ml^−1^ Benzonase) with a mixture of protease inhibitor^57^, incubated for 30 min at 4 °C and these lysates were used as whole-cell lysates. Protein concentration was measured with the Bio-Rad Protein Assay (Bio-Rad). Samples were mixed with 4 × Laemmli's SDS loading buffer (Boston Bioproducts), boiled at 95 °C for 5 min and subjected to SDS–PAGE. After proteins were transferred to a nitrocellulose membrane (Bio-Rad), the membranes were blocked in Tris-buffered saline containing 0.1% (v/v) Tween 20 and 5% (w/v) nonfat dry milk for 1 h at room temperature. Immunoblots were carried out with the antibodies described in Supplementary Table 1, and visualized using the ECL Western Blotting Detection Reagents (GE Healthcare). For stripping off antibody, OneMinute Plus Western Blot Stripping Buffer (GM Biosciences) was used according to the manufacturer's instruction. Signal intensity was quantified using the ImageJ software (National Institutes of Health). Uncropped images of all blots are provided in Supplementary Fig. 9.

### Expression plasmids

pLKO.1-based plasmids for shRNAs were obtained from the RNAi Consortium (Broad Institute). The RNAi Consortium clone ID and target sequence of each vector are provided in Supplementary Table 2. The human *KDM3A* cDNA was amplified using PCR and ligated into the HpaI and XhoI sites of pMSCV retroviral expression vector (Clontech). The human *KLF2* cDNA was amplified using PCR and ligated into pCR-Blunt (Invitrogen), and then cloned into the EcoRI site of pcDNA3 (Invitrogen) or pMSCV retroviral expression vector (Clontech). The human *IRF4* cDNA was amplified using PCR and ligated into the EcoRI and XhoI sites of pMSCV retroviral expression vector (Clontech). To generate shRNA-resistant cDNAs, six point mutations (silent mutations) were introduced into shRNA-targeting sequences of *KDM3A* and *KLF2* with the QuikChange II Site-Directed Mutagenesis kit (Agilent Technologies). Cloning and mutagenesis primers used in this study are shown in Supplementary Tables 3 and Supplementary Table 4.

### Viral production and infection

On day 0, 293T packaging cells were plated at a density of 6 × 10^5^ cells per six-well plates. On day 1, cells were transfected with 500 ng of pLKO.1 plasmid, 500 ng of pCMV-dvpr and 100 ng of VSV-G for lentivirus packaging, or were transfected with 500 ng of pMSCVneo plasmid, 500 ng of pMD-MLV and 100 ng of VSV-G for retrovirus packaging, using TransIT-LT1 Transfection Reagent (Mirus), according to the manufacturer's instructions. On day 2, media were replaced and cells were cultured for an additional 24 h to obtain viral supernatants. On day 3, media containing virus were harvested, passed through 0.45-μm cellulose acetate membrane filters and used fresh for infection. Overall, 1 × 10^6^ cells per 1 ml of crude viral supernatants in the presence of 8 μg ml^−1^ polybrene were spinoculated at 800*g* for 30 min at room temperature, and then incubated in 5% CO_2_ at 37 °C for 5 h. Media were then replaced. After 24 h of viral infection, cells expressing shRNAs were selected with puromycin dihydrochloride (Sigma-Aldrich) at 1 μg ml^−1^ for 2 days, and then subjected to each assay. Cells expressing cDNAs were selected with G418 disulfate (Sigma-Aldrich) at 600 μg ml^−1^ for at least 7 days, and clones stably expressing cDNAs were subjected to rescue experiments. Puromycin and G418 concentrations were titrated to identify the minimum concentration of each drug that caused complete cell death of uninfected cells after 2 or 7 days, respectively.

### RNA extraction and QRT–PCR

Total RNA was extracted using the RNeasy Mini Kit (Qiagen). cDNA was synthesized from 2 μg of total RNA with oligo(dT) primers using the SuperScript III First-Strand Synthesis System (Invitrogen). Real-time PCR was performed in 96-well plates using the Applied Biosystems 7300 or 7500 Real-Time PCR System (Applied Biosystems). The PCR mixture contained 20 ng of reverse-transcribed RNA, 200 nM of forward and reverse primers and SYBR green PCR Master Mix (Applied Biosystems) in a final volume of 20 μl. The conditions were 95 °C for 10 min, followed by 40 cycles of 15 s at 95 °C and 1 min at 60 °C. The relative amount of each transcript was calculated using the relative standard curve method. *Cyclophilin A* mRNA was used as the invariant control, and values were normalized by *Cyclophilin A* expression. Specific primers for each gene transcript are shown in Supplementary Table 5.

### Cell growth assay

Cell growth was assessed by measuring 3-(4,5-Dimethyl-2-thiazolyl)-2,5-diphenyl-2H-tetrazolium bromide (MTT, Sigma-Aldrich) dye absorbance^58^. Cells cultured in 96-well plates (100 μl per well) were pulsed with 10 μl of 5 mg ml^−1^ MTT for the last 4 h of cultures, followed by addition of 100 μl of isopropanol containing 0.04 N HCl. Absorbance was measured at 570 nm, with 630 nm as a reference wavelength, using a spectrophotometer (SpectraMax M3, Molecular Devices).

DNA synthesis was measured by ^3^H-thymidine (PerkinElmer) uptake^58^. Cells incubated in 96-well plates were pulsed with ^3^H-thymidine (0.25 Ci per well) during the last 5 h of cultures. Cells were then harvested on fibreglass filters (Filtermat A, PerkinElmer) with Tomtec Cell Harvester, and ^3^H-thymidine incorporation was measured using 1450 MicroBeta TriLux scintillation counter (PerkinElmer).

### Subcutaneous xenograft model

Nine-week-old male CB17 SCID mice (Charles River Laboratories) were used for this study. Overall, 4 × 10^6^ viable MM.1S cells transduced with the corresponding shRNA (shKDM3A, shKLF2 or shLuc) were suspended in 100 μl of PBS and then inoculated subcutaneously into the right flank of 200-cGy-irradiated mice. Tumour growth was monitored twice a week using an electronic caliper, and tumour volume was calculated using the formula: (length × width^2^) × 2^−1^, where length is greater than width. Animal studies were performed under a protocol approved by the Dana-Farber Institutional Animal Care and Use Committee.

### Apoptosis assay

Quantification of apoptotic cells was performed by Apo2.7 immunostaining, which specifically detects the mitochondrial membrane antigen exposed on cells undergoing apoptosis^59^. Overall, 5 × 10^5^ cells were incubated with 100 μg ml^−1^ of digitonin (Sigma-Aldrich) in 100 μl of PBS with 2.5% (v/v) fetal bovine serum (PBSF) for 20 min at 4 °C to permeabilize cells. After washing with 2 ml of PBSF, cells were incubated with 20 μl of anti-Apo2.7-R-phycoerythrin (PE) or mouse IgG_1_ isotype control-PE (Beckmann Coulter) in 100 μl of PBSF for 15 min at room temperature in the dark. After washing with 2 ml of PBSF, cells were suspended with 1 ml of PBSF and then analysed with flow cytometry (BD FACSCanto II, BD Biosciences). The proportion of apoptotic cells was assessed by measuring PE. In an optimization experiment with IgG_1_ isotype control, the possibility of nonspecific background was excluded.

### Cell cycle assay

Cells were harvested and fixed with 70% ethanol for 20 min on ice. After washing with PBS twice, cells were incubated with 5 μg ml^−1^ RNase (Roche) in PBS for 20 min at room temperature, and then resuspended in PBS containing 10 μg ml^−1^ propidium iodide (Sigma-Aldrich). The stained cells were analysed with flow cytometry (BD FACSCanto II, BD Biosciences), and the percentage of cells in G1, S and G2/M phases was determined using the ModFit LT software (Verity Software House).

### ChIP assay

ChIP assay was performed using the SimpleChIP Plus Enzymatic Chromatin IP Kit (Cell Signaling), according to the manufacturer's protocol. For ChIP with anti-KDM3A, ∼5 × 10^6^ RPMI8226 cells were crosslinked with 1.5 mM ethylene glycolbis (succinimidylsuccinate) in PBS for 20 min at room temperature and then crosslinked with 1% formaldehyde for 10 min at room temperature. For ChIP with anti-methylated histone lysine, anti-KLF2 and anti-IRF4, 5–10 × 10^6^ RPMI8226 cells were crosslinked with 1% formaldehyde for 10 min at room temperature. After quenching the reaction with glycine solution, samples were washed twice with ice-cold PBS, resuspended and incubated with micrococcal nuclease for 20 min at 37 °C. After stopping DNA digestion by adding 50 mM EDTA, nuclear pellets were resuspended and sonicated to break nuclear membranes using Bioruptor (Diagenode) at power level *H* for 15 × 30-s pulses (30-s pause between pulses). After clarifying lysates by centrifugation, supernatants were incubated overnight at 4 °C with antibodies described in Supplementary Table 1, followed by incubation with Protein G agarose beads for 2 h. Beads were washed four times, and immune complexes were eluted by heating at 65 °C, with occasional vortexing. Crosslinking was then reversed by incubation overnight at 65 °C. After treating with proteinase K, DNAs were purified with purification spin column. The amount of DNA recovered from immunoprecipitates with specific antibodies or control IgG was determined with quantitative PCR using Applied Biosystems 7300 or 7500 Real-Time PCR system, which was performed in triplicate. Relative occupancy values were determined by calculating the ratios of the amount of immunoprecipitated DNA to that of the input sample. For ChIP with anti-KDM3A, anti-KLF2 and anti-IRF4, the values were normalized to the values of control IgG, defined as 1.0. For ChIP with anti-methylated histone lysine, data are shown as fold enrichment compared with sh control (shLuc) samples. Specific primers for each amplified region are shown in Supplementary Table 6.

### Affymetrix transcriptome analysis

Total RNAs for microarray analyses were extracted from RPMI8226 cells transduced with shRNA targeting *KDM3A* or *KLF2*, or control shRNA in biological duplicate with the RNeasy Mini kit (Qiagen). Total RNA (1 μg) was processed, and labelled cRNA was hybridized to Human Genome U133 plus 2.0 arrays (Affymetrix), according to the standard Affymetrix protocols. U133 plus 2.0 is a most comprehensive array that contains >54,000 probe sets representing ∼38,500 genes. Expression data can be found at http://www.ncbi.nlm.nih.gov/geo/ under accession number GSE55667.

*Quality control and normalization of transcriptome data*. The quality of scanned array images was determined on the basis of background values, RNA degradation plot, array intensity distribution plots, MA plots, per cent present calls, scaling factors and 3′–5′ ratio of β-actin and GAPDH using various bioconductor packages for R (ref. 60). The normalized transcript expression values were generated from high-quality array images using the Robust Multichip Average algorithm in R using the Bioconductor and associated packages. Robust Multichip Average performed background adjustment, quantile normalization and final summarization of oligonucleotides per transcript using the median polish algorithm^61^. To identify the outlier arrays, unsupervised analysis was performed using principal component analysis and hierarchical clustering. The outlier arrays were deleted from further supervised analysis. Transcripts were filtered to include only those with absolute expression ≥40 in at least 10% of samples to reduce the false-positive results.

*Identification of differentially expressed genes*. To identify an accurate signature for target gene knockdown, we performed knockdown using two different shRNAs. Initially individual shRNA signatures of differentially expressed transcripts were generated by comparing control versus target-gene shRNA samples for each shRNA. The transcripts with a 90% lower confidence bound (LCB) of the fold change between the two groups above 1.5 were considered to be differentially expressed^62^. LCB is a stringent estimate of fold change and has been shown to be the better ranking statistic^63^. Further, a consensus gene knockdown signature was generated from individual shRNA signatures by including genes that were consistently up- or downregulated by at least 1.15 LCB in both shRNAs and an average LCB greater than 1.5.

### Gene set enrichment analysis

GSEA was performed using the GSEA-R, a bioconductor implementation of GSEA from Broad Institute^64^. The GSEA analysis was performed to compare transcriptome signature of *KDM3A* knockdown with *KLF2* knockdown and *vice versa*, or transcriptome signature of *IRF4* knockdown with *KLF2* knockdown. Before GSEA analysis, normalization of expression data and generation of transcriptome signature were performed using an approach described in the previous section. Transcriptome signature of *IRF4* knockdown was obtained from previous report^29^. Analysis was run with 1,000 permutations and a classic statistic; NES, normalized enrichment score and nominal *P* value was measured.

### Construction of the promoter reporter gene

pIRF4(1200) is the human *IRF4* promoter-luciferase reporter construct that spans positions −1,200 to +14 relative to the transcription start site. pIRF4(520), pIRF4(480) and pIRF4(160) contain deletion constructs with the 5′ end noted in parentheses, and the same 3′ end point at +14. The 5′ flank region of the human *IRF4* was amplified with PCR using genomic DNA isolated from RPMI8226 cells as a template. Then, the digested PCR product was cloned into the XhoI–EcoRV sites of pGL4.10[luc2] (Promega). Base substitution mutants were generated in pIRF4(520) with the QuikChange II Site-Directed Mutagenesis kit (Agilent Technologies), according to the manufacturer's protocol. The details of the primers are shown in Supplementary Tables 3 and Supplementary Table 4.

### Luciferase reporter assay

293T cells were plated at a density of 1.2 × 10^5^ per 24-well plate on the day before transfection. Cells were transfected with luciferase reporter plasmid (0.2 μg), the indicated amount of expression plasmid and pSV-β-Gal (Promega; 0.1 μg) with TransIT-LT1 Transfection Reagent (Mirus), according to the manufacturer's instructions. The total amount of DNA per well was kept constant by adding the empty vector, which has the same vector backbone of expression plasmid. After 24 h, the cells were washed with PBS and lysed in 100 μl of Reporter Lysis Buffer (Promega). The suspension was incubated for 15 min at room temperature and centrifuged at top speed in a microfuge for 1 min. The supernatant was used for firefly luciferase activity assay. Values are normalized to β-galactosidase activity^65^.

### MM cell adhesion assay

Bone marrow stromal cells were plated at a density of 1 × 10^4^ per well in a 96-well plate 1 day before the adhesion assay. Human fibronectin- or BSA-coated plates were purchased from R&D Systems. MM cells were prelabelled by incubating in the presence of 1 μM calcein AM (Molecular Probes) at 37 °C for 30 min. After washing with PBS with calcium and magnesium (PBS(+)) twice, labelled cells were resuspended in PBS(+) at a concentration of 1 × 10^6 ^ml^−1^. Overall, 1 × 10^5^ cells were then incubated in 96-well plates coated with bone marrow stromal cells, fibronectin or BSA at 37 °C for 2 h. After measuring the fluorescent intensity of pre-wash samples, wells were washed two to three times with PBS. The fluorescent intensity of adherent cells was then quantified using a fluorescence plate reader (SpectraMax M3, Molecular Devices) at an excitation wavelength of 485 nm and an emission wavelength of 525 nm. Per cent adhesion was determined by calculating the ratios of the fluorescent intensity of post-wash sample to that of pre-wash sample.

### Transwell migration assay

For migration assay, 4 × 10^5^ MM.1S cells suspended in 200 μl of RPMI1640 medium with 1% fetal bovine serum were placed in the upper chambers, and 500 μl of RPMI1640 medium with 1% fetal bovine serum with CXCL12 (20 nM) were placed in the lower chambers of 24-well transwell plates (pore size 8.0 μm; Costar). After 4-h incubation at 37 °C, cells migrated to the lower chambers were labelled with calcein AM (Molecular Probes) at 37 °C for 1 h and were quantified using a fluorescence plate reader (SpectraMax M3, Molecular Devices), at an excitation wavelength of 485 nm and emission wavelength of 525 nm. Overall, 4 × 10^5^ MM.1S cells were also labelled and quantified as input. Per cent migration was determined by calculating the ratios of the fluorescent intensity of migrated cells to that of input cells.

### MM cell homing assay

MM.1S-Luc-GFP cells stably expressing the corresponding shRNA (shKDM3A, shKLF2, shIRF4 or control shRFP; 15 × 10^6^ viable cells) were injected via tail vein into 300-cGy-irradiated NOD-SCID male mice (Charles River Laboratories). Twenty-four hours after injection, mice were killed, and the bilateral femurs of each mouse were collected. After crunching femurs, bone marrow cells are sieved through a 70-μm filter, and red blood cells were lysed with ACK Lysing Buffer (Lonza). The bone marrow cells were then resuspended in PBS with 2% (v/v) fetal bovine serum, and GFP-positive cells were detected with flow cytometry (BD FACSCanto II, BD Biosciences). Data are shown as relative homing efficacy compared with control cells expressing shRFP. Animal studies were performed under a protocol approved by the Dana-Farber Institutional Animal Care and Use Committee.

### Statistical analysis

Student's *t*-test or analysis of variance followed by Dunnett's test was used to compare differences between the treated group and relevant control group. A value of *P*<0.05 was considered significant.

## Additional information

**Accession codes:** The microarray data have been deposited in the Gene Expression Omnibus database under accession code GSE55667.

**How to cite this article:** Ohguchi, H. *et al.* The KDM3A–KLF2–IRF4 axis maintains myeloma cell survival. *Nat. Commun.* 7:10258 doi: 10.1038/ncomms10258 (2016).

## Supplementary Material

Supplementary Information Supplementary Figures 1-9, Supplementary Tables 1-6 and Supplementary References.

Supplementary Data 1 Genes down regulated and up regulated by KDM3A knockdown in RPMI8226 cells.

Supplementary Data 2 Genes down regulated and up regulated by KLF2 knockdown in RPMI8226 cells.
