# Supplementary material for: The KDM3A–KLF2–IRF4 axis maintains myeloma cell survival
Source: Nat Commun. 2016 Jan 5;7:10258. doi: 10.1038/ncomms10258 (PMC4728406; doi:10.1038/ncomms10258)
Supplement: Supplementary Information — Supplementary Figures 1-9, Supplementary Tables 1-6 and Supplementary References. [file ncomms10258-s1.pdf]

Supplementary Information

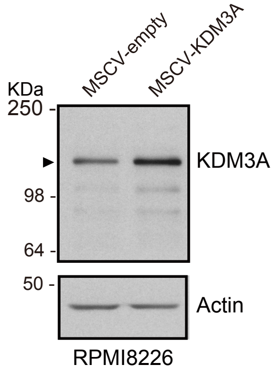

Supplementary Figure 1 | Overexpression of *KDM3A* in RPMI8226 cells. Protein level of KDM3A in RPMI8226 cells expressing the *KDM3A* cDNA. RPMI8226 cells were transduced with either the *KDM3A* cDNA or empty vector by retrovirus. Whole cell lysates were subjected to immunoblot analysis with KDM3A and Actin antibodies. Data are representative of at least three independent experiments.

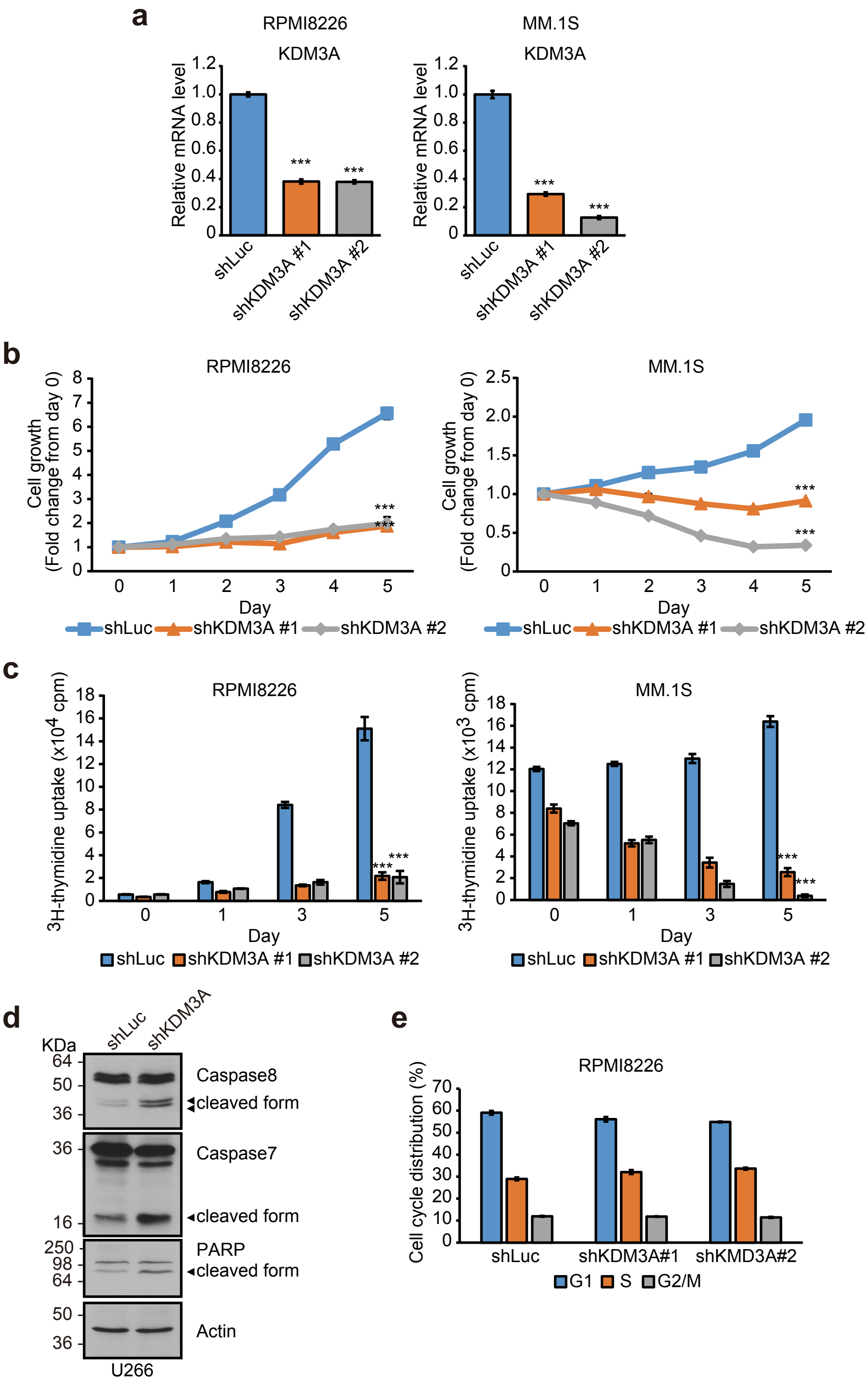

Supplementary Figure 2 | Knockdown of *KDM3A* in MM cells. (**a**) Quantification of *KDM3A* mRNA levels in RPMI8226 and MM.1S cells transduced with either *KDM3A*-specific shRNA (shKDM3A #1 and #2) or control shRNA targeting

luciferase (shLuc) by lentivirus. Values represent the amount of mRNA relative to shLuc, which is arbitrarily defined as 1. Data represent mean ± s.d. of triplicate measurements. (**b,c**) Significant reduction of cell growth and DNA synthesis after silencing of *KDM3A* in MM cells. RPMI8226 or MM.1S cells were transduced with either shKDM3A or shLuc. Three days post-infection, which was designated as day 0, viable cells were counted by trypan blue exclusion and plated in 96-well plates. Cells were cultured for indicated intervals from day 0, and analyzed for growth by MTT assay (**b**) or analyzed for DNA synthesis using ^{3}H-thymidine uptake (**c**). Cell growth is shown as fold change compared to day 0 (**b**). Data represent mean ± s.d. of quintuplicate cultures. (**d**) Immunoblot analysis of caspase 8, caspase 7, and PARP in whole cell lysates from *KDM3A*-knockdown U266 cells. Arrowheads indicate cleaved form. (**e**) Cell cycle distribution in *KDM3A*-knockdown RPMI8226 cells. RPMI8226 cells transduced with either shKDM3A or shLuc (4 days post-infection) were analyzed for cell cycle distribution by staining with propidium iodide using a flow cytometer. Data represent mean ± s.d. of duplicate measurements. For (**a-e**), data are representative of at least two independent experiments. ***P < 0.001 compared with control; Student's *t*-test.

a

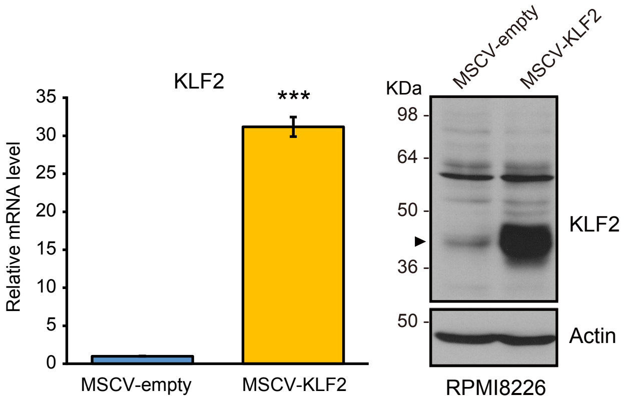

b

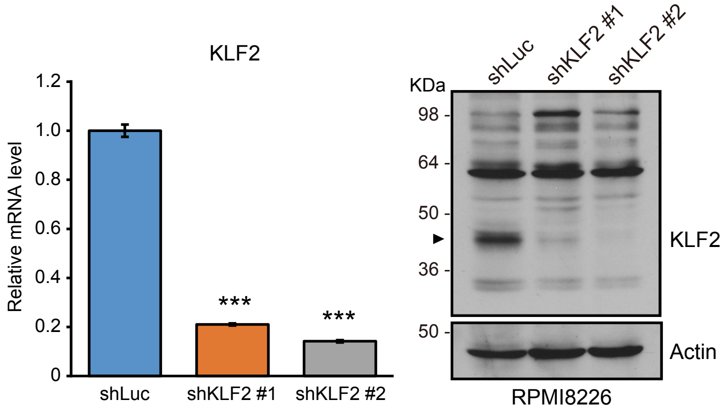

Supplementary Figure 3 | Overexpression and knockdown of *KLF2* in RPMI8226 cells. (**a**) Quantification of *KLF2* mRNA and protein levels in RPMI8226 cells expressing the *KLF2* cDNA. RPMI8226 cells were transduced with either the *KLF2* cDNA or empty vector by retrovirus. Cells were harvested for isolation of total RNA or whole cell lysates, and subjected to quantitative real time PCR or immunoblot analysis with KLF2 and Actin antibodies. (**b**) Quantification of *KLF2* mRNA and protein levels in RPMI8226 cells transduced with either *KLF2*-specific shRNA (shKLF2 #1 and #2) or control shRNA targeting *luciferase* (shLuc) by lentivirus. For (**a,b**), data represent mean ± s.d. of triplicate measurements (left panel), and are representative of at least two independent experiments.

***P < 0.001 compared with control; Student's *t*-test.

a

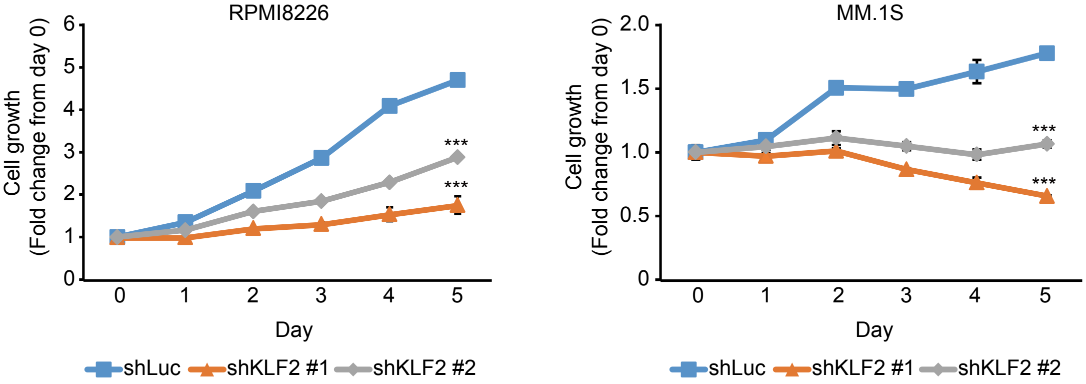

b

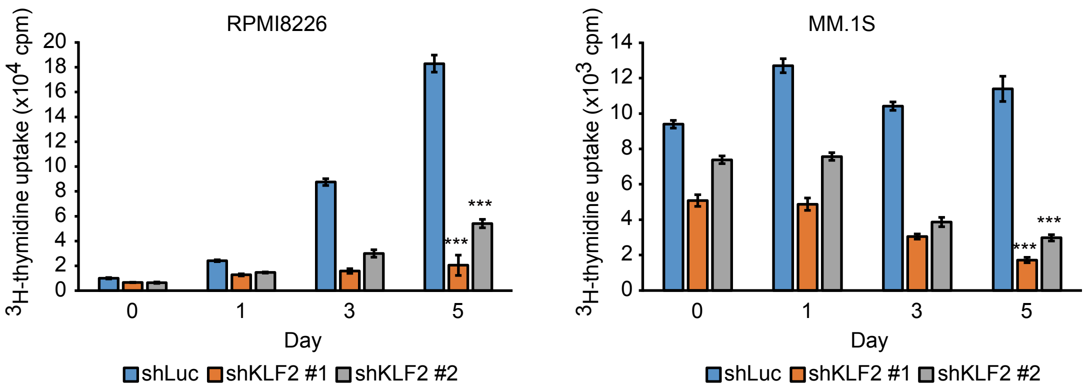

Supplementary Figure 4 | Knockdown of *KLF2* significantly reduces cell growth and DNA synthesis in MM cells. (**a**,**b**) RPMI8226 or MM.1S cells were transduced with either *KLF2*-specific shRNA (shKLF2 #1 and #2) or control shRNA (shLuc) by lentivirus. Cells were cultured for indicated intervals from day 0 (three days post-infection), and analyzed for growth by MTT assay (**a**) or analyzed for DNA synthesis using ^{3}H-thymidine uptake (**b**). Cell growth is shown as fold change compared to day 0 (**a**). Data represent mean ± s.d. of quintuplicate cultures, and are representative of two independent experiments. ***$P < 0.001$ compared with control; Student's *t*-test.

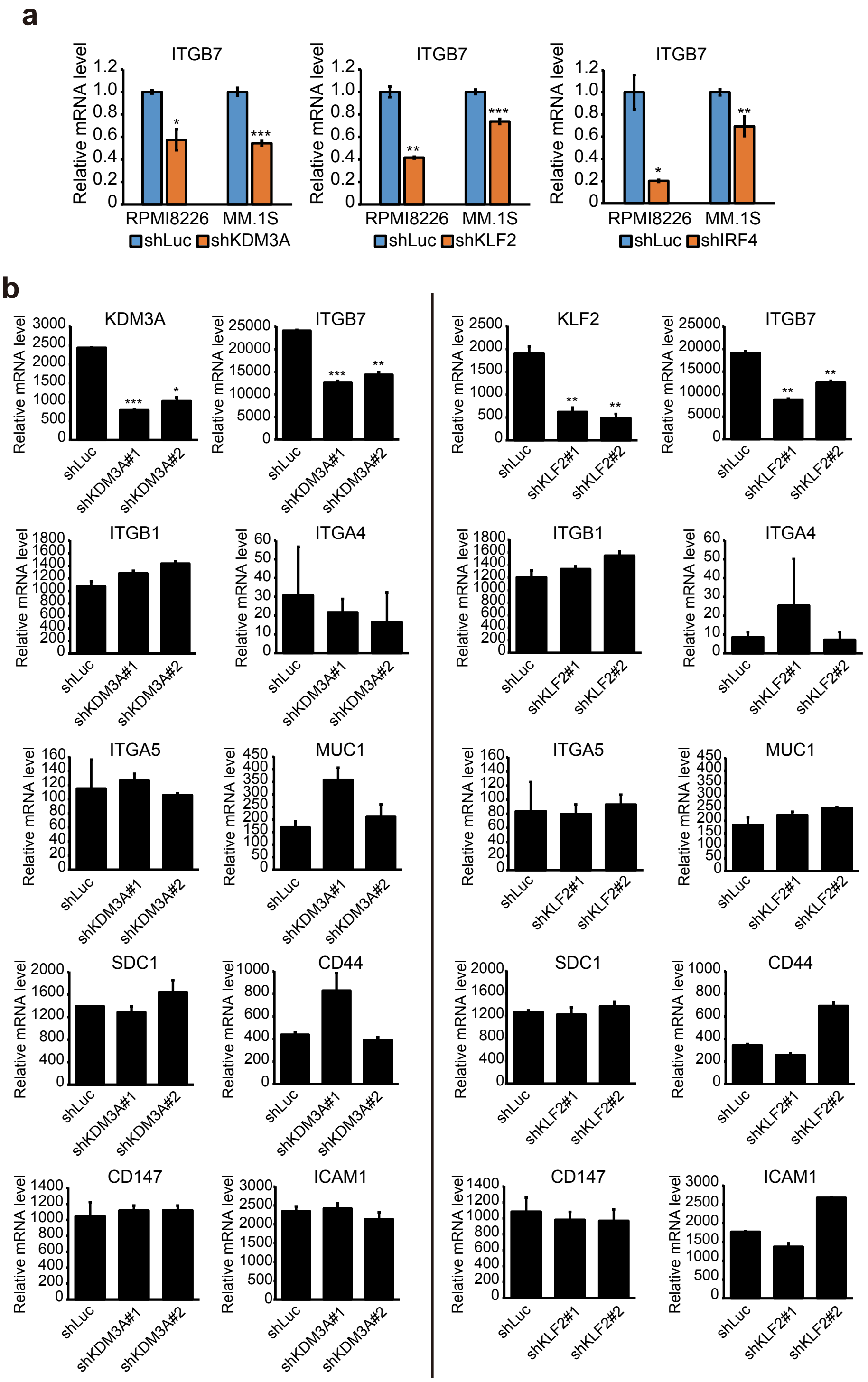

Supplementary Figure 5 | Expression of *ITGB7* and other adhesion molecules after knockdown of *KDM3A* or *KLF2*. (**a**) Quantification of *ITGB7* mRNA levels in RPMI8226 and MM.1S cells transduced with either shRNA against *KDM3A*

(shKDM3A), *KLF2* (shKLF2), *IRF4* (shIRF4), or control shRNA (shLuc). Cells were harvested for isolation of total RNA and subjected to quantitative real time PCR. Data represent mean ± s.d. of triplicate measurements. (**b**) Microarray data in RPMI8226 cells transduced with shKDM3A, shKLF2, or control shLuc were analyzed for mRNA expression of *ITGB7*, *ITGB1*, *ITGA4*, *ITGA5*, *MUC1*, *SDC1*, *CD44*, *CD147* and *ICAM1*. Left panel; shKDM3A dataset, right panel; shKLF2 dataset. Data represent mean ± s.d. of duplicate experiments. *$P < 0.05$, **$P < 0.01$, ***$P < 0.001$ compared with control; Student's *t*-test.

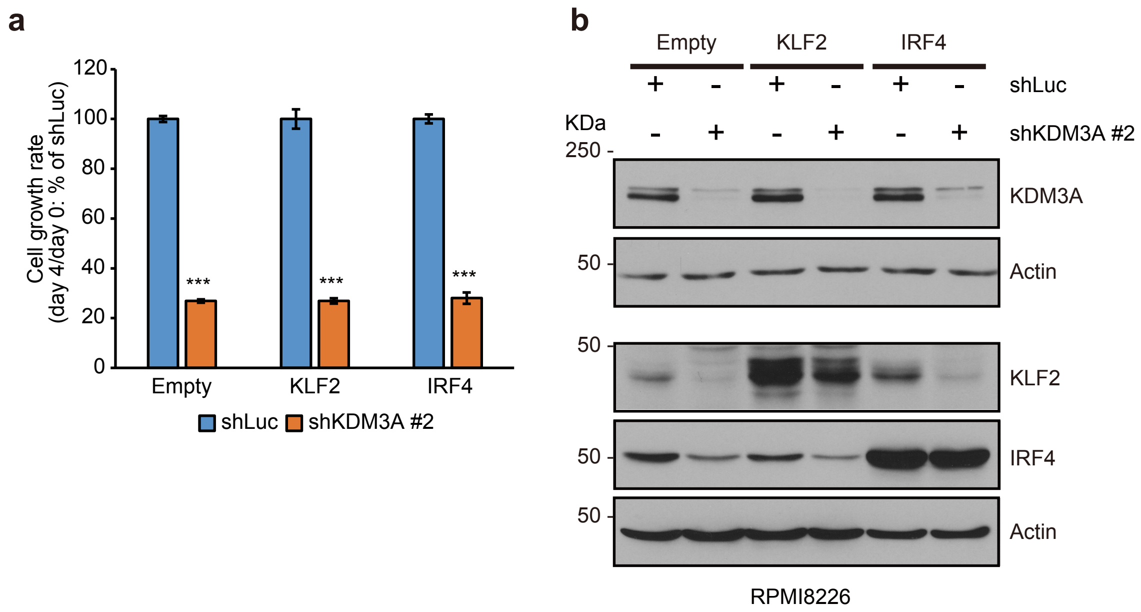

Supplementary Figure 6 | Knockdown of *KDM3A* in *KLF2* or *IRF4*-overexpressed RPMI8226 cells. (**a**) RPMI8226 cells were retrovirally transduced with the *KLF2* or *IRF4* cDNA, or empty vector. Cells expressing the *KLF2* or *IRF4* cDNA, or empty vector were then lentivirally transduced with shKDM3A or shLuc. Cell growth rate (day 4/day 0) after lentiviral infection was determined for shKDM3A relative to shLuc. The growth rate for control shLuc in each cells expressing the *KLF2* or *IRF4* cDNA, or empty vector is set as 100%, respectively. Data represent mean ± s.d. of quintuplicate cultures. ***$P < 0.001$ compared with control; Student's *t*-test. (**b**) Immunoblot analysis of KDM3A, KLF2, or IRF4 after knockdown of *KDM3A* in *KLF2* or *IRF4*-overexpressed RPMI8226 cells. Actin served as the loading control for each membrane. For (**a,b**), data are representative of two experiments.

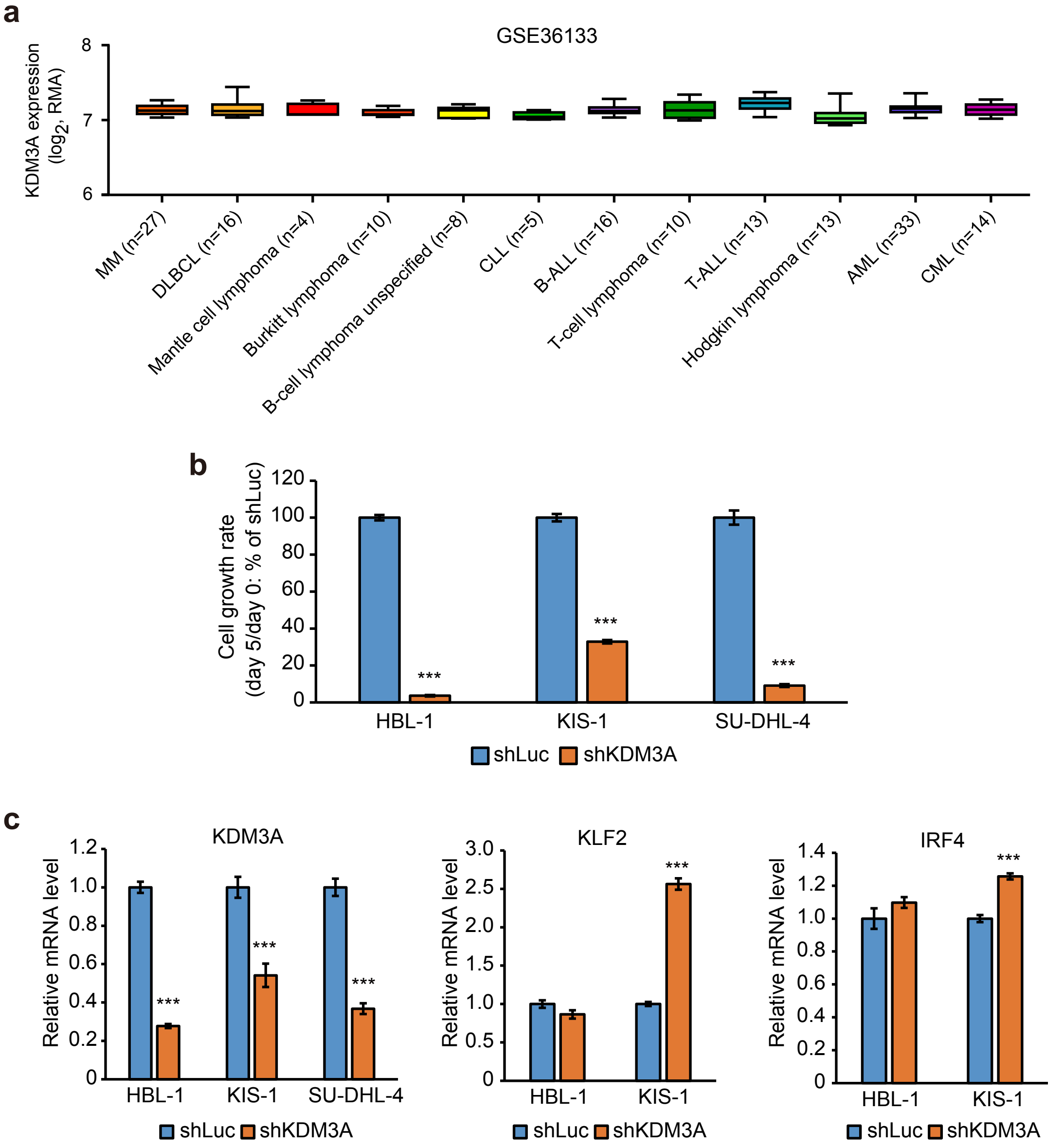

Supplementary Figure 7 | Knockdown of *KDM3A* in DLBCL cell lines. (**a**) Microarray data set (GSE36133) was analyzed for mRNA expression of *KDM3A* in hematologic cancer cell lines. MM; multiple myeloma, DLBCL; diffuse large B cell lymphoma, CLL; chronic lymphocytic leukemia, B-ALL; B-cell acute lymphoblastic leukemia, T-ALL; T-cell acute lymphoblastic leukemia, AML; acute myelogenous leukemia, CML; chronic myelogenous leukemia. (**b**) Effect of *KDM3A* knockdown on DLBCL cell growth. DLBCL cell lines (HBL-1, KIS-1, and SU-DHL-4) were

transduced with shKDM3A or control shLuc. Three days post-infection (after 2 days of puromycin selection), which was designated as day 0, cells were plated in 96-well plates. Cell viability was measured on day 0 and day 5 by MTT assay, and cell growth rate (day 5/day 0) relative to shLuc was determined. Data represent mean ± s.d. of quintuplicate cultures. ***$P < 0.001$ compared with control; Student's *t*-test. (**c**) Quantitative real time PCR of *KDM3A*, *KLF2* and *IRF4* in HBL-1, KIS-1, and SU-DHL-4 cells transduced with either shKDM3A or shLuc. After 3 days of infection, cells were harvested for isolation of total RNA. Values represent the amount of mRNA relative to control shLuc sample, defined as 1. *KLF2* and *IRF4* expression of SU-DHL4 is not shown because *KLF2* and *IRF4* expression is almost undetectable in this cell line. Error bars represent s.d. of triplicate measurements. For (**b,c**), data are representative of two experiments. ***$P < 0.001$ compared with control; Student's *t*-test.

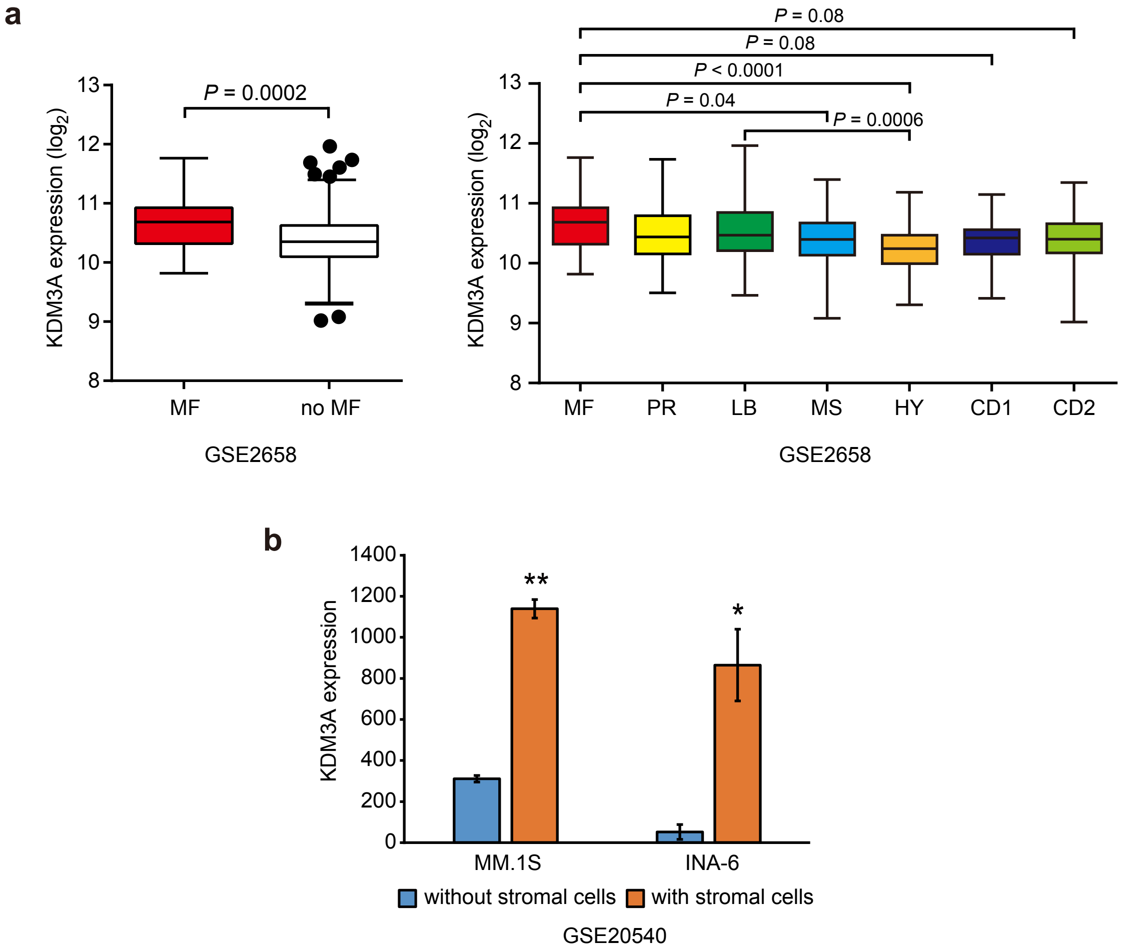

Supplementary Figure 8 | *KDM3A* expression in MM cells. (**a**) *KDM3A* expression is elevated in MM patient samples related to *MAF* translocations and high *MAF* expression. Microarray data set (GSE2658) was analyzed for mRNA expression of *KDM3A* in newly diagnosed MM patient samples. MF, PR, LB, MS, HY, CD1, and CD2 are the molecular classified subgroups defined by Zhan et al.[1]. MF is the subgroup associated with *MAF* translocations and high *MAF* expression. *P* values were determined by Student's *t*-test (left panel), or ANOVA followed by Tukey's test (right panel). (**b**) *KDM3A* expression in MM.1S and INA-6 cells cultured alone or with HS-5 bone marrow stromal cells. Publicly available microarray data set (GSE20540) was analyzed for mRNA expression of *KDM3A* in MM cells. Data represent mean ± s.d. of duplicate measurements. *$P < 0.05$, **$P < 0.01$ compared with control; Student's *t*-test.

Fig. 1b

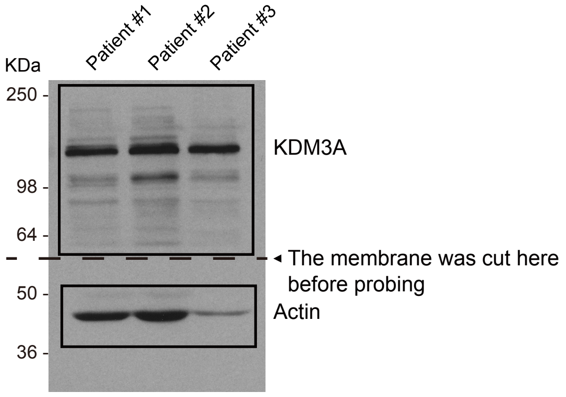

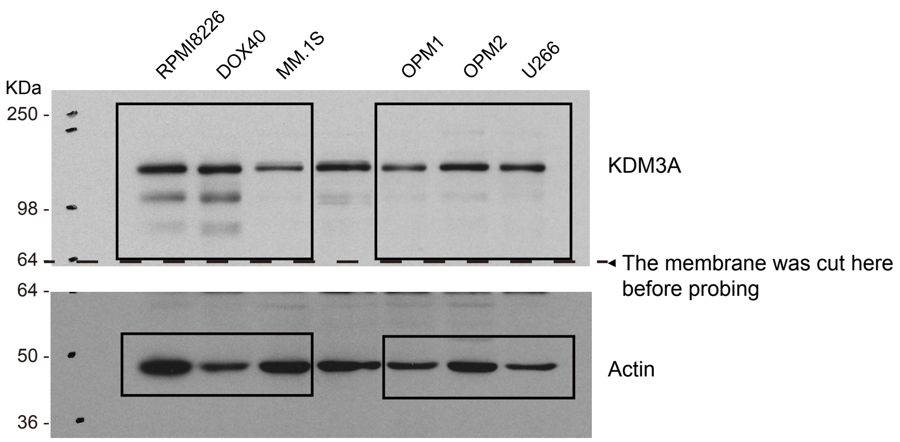

Supplementary Figure 9 | Original images of immunoblot analyses.

Fig. 2

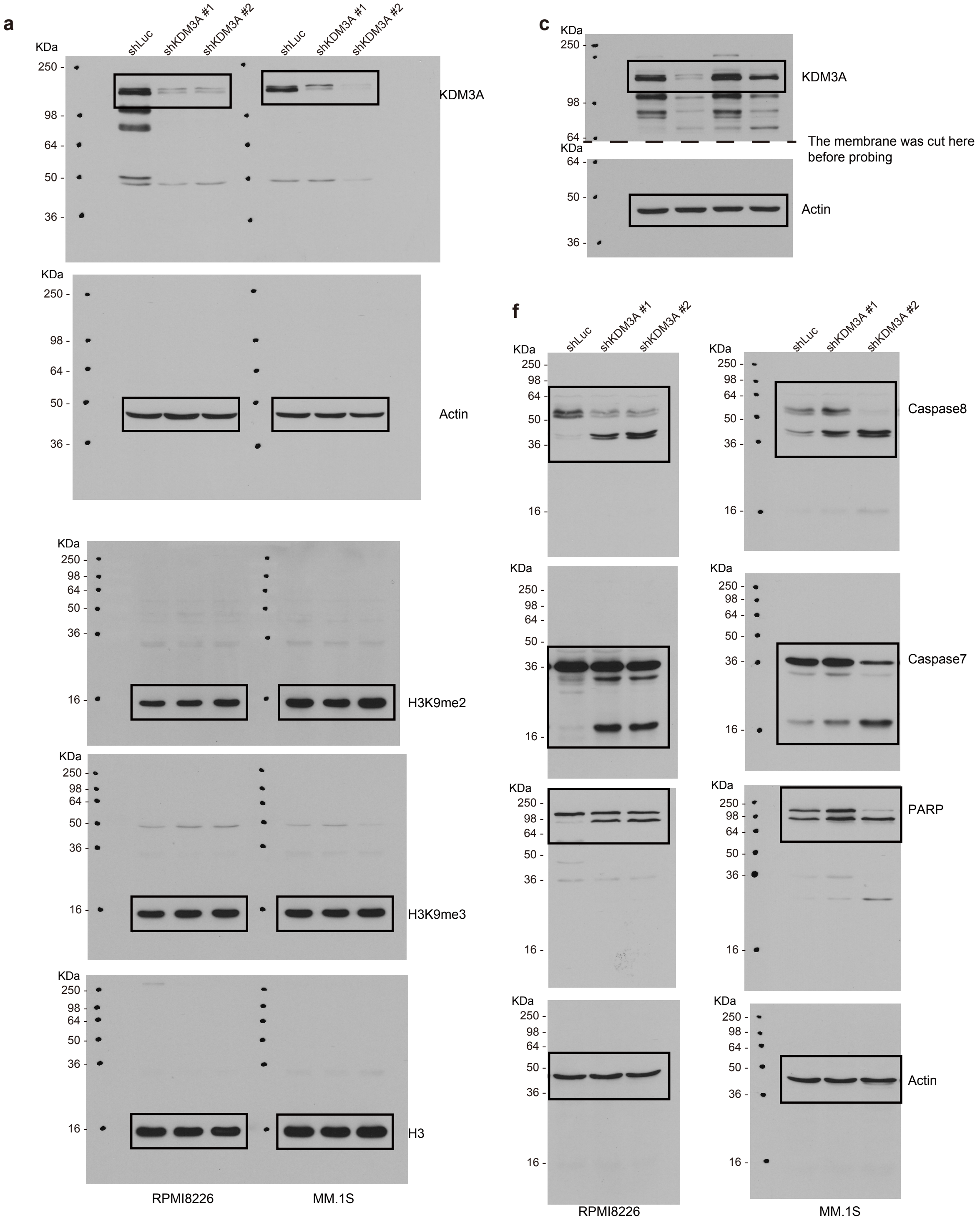

Supplementary Figure 9 | Original images of immunoblot analyses.

Fig. 3

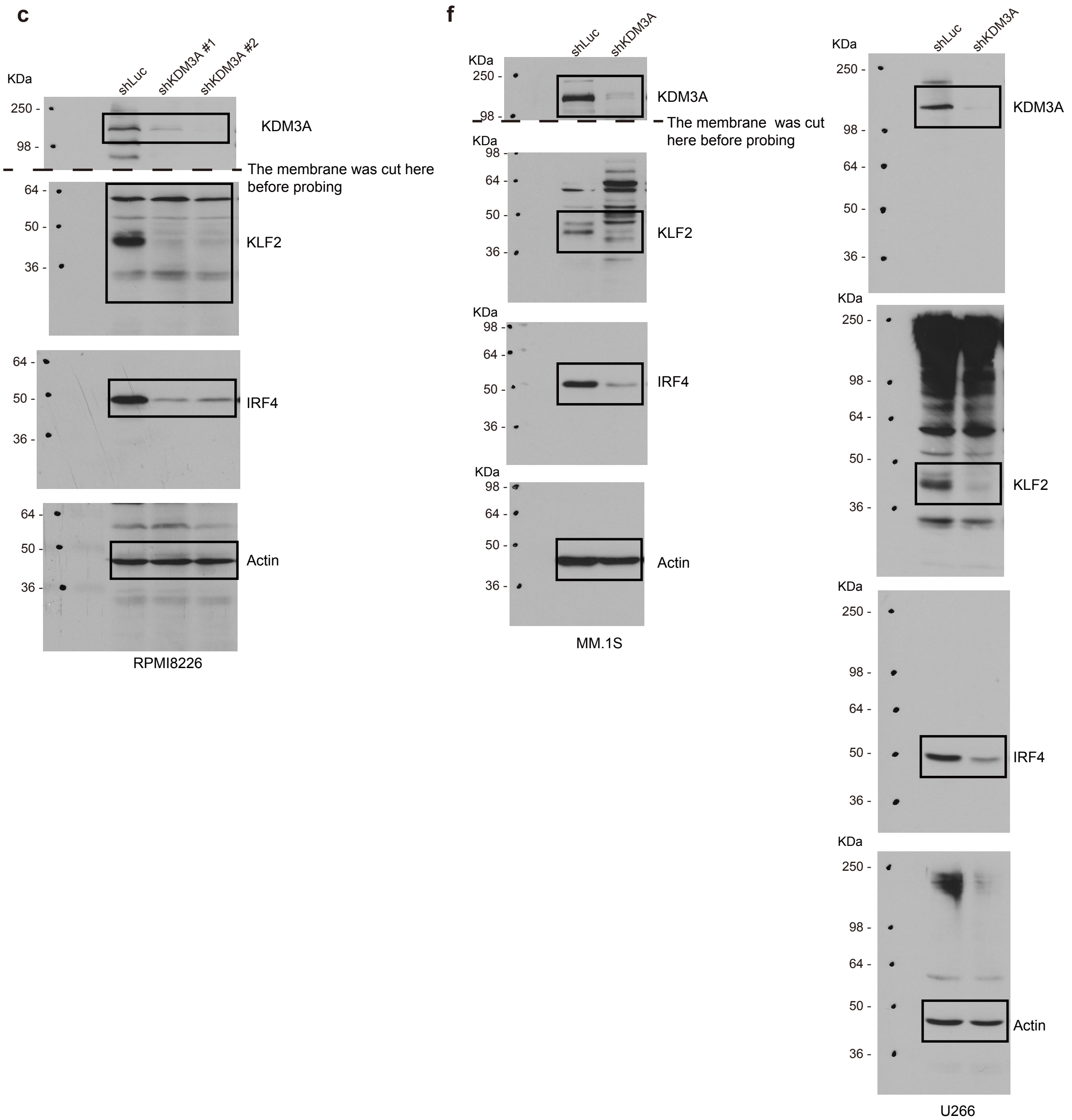

Supplementary Figure 9 | Original images of immunoblot analyses.

Fig. 4

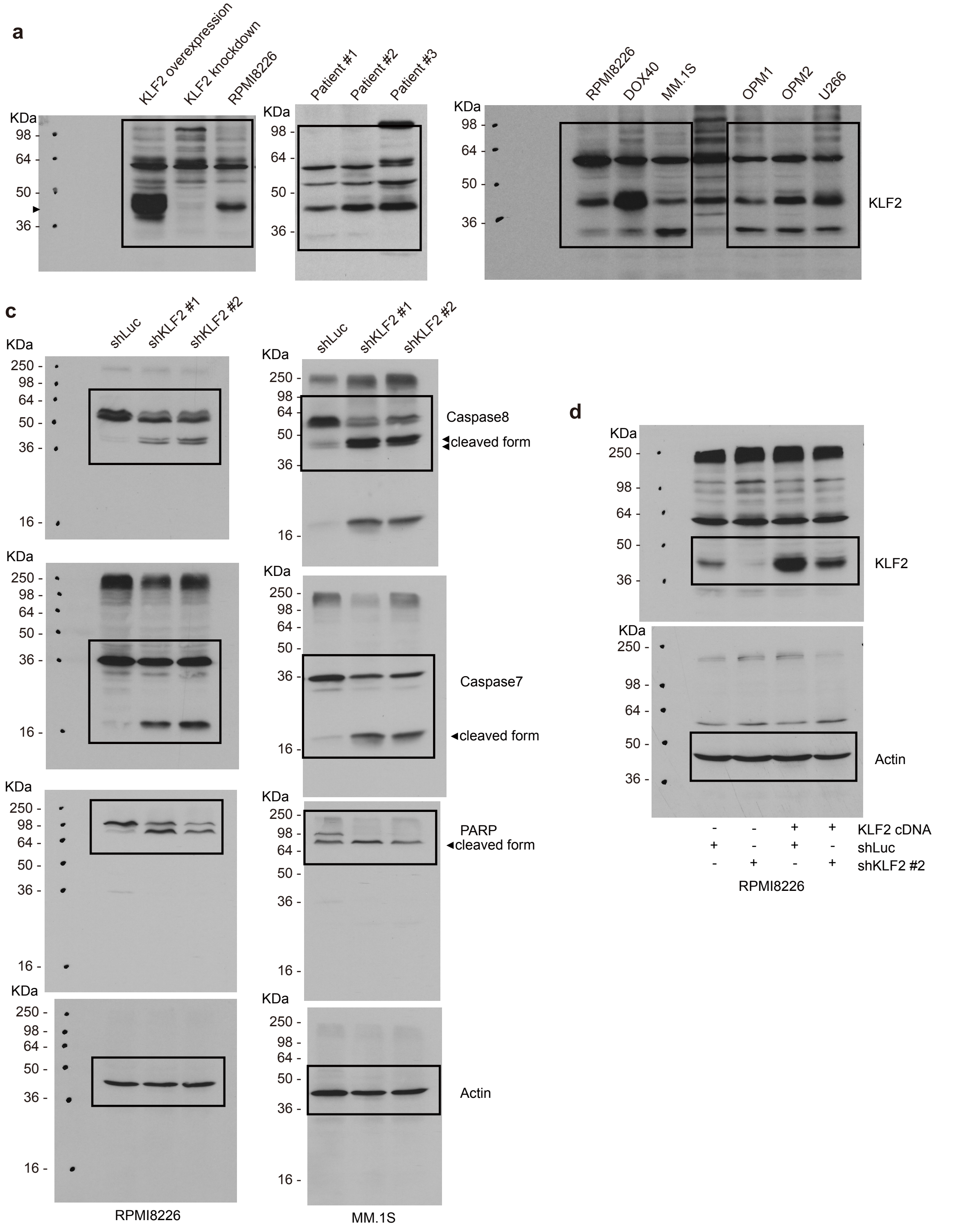

Supplementary Figure 9 | Original images of immunoblot analyses.

Fig. 5

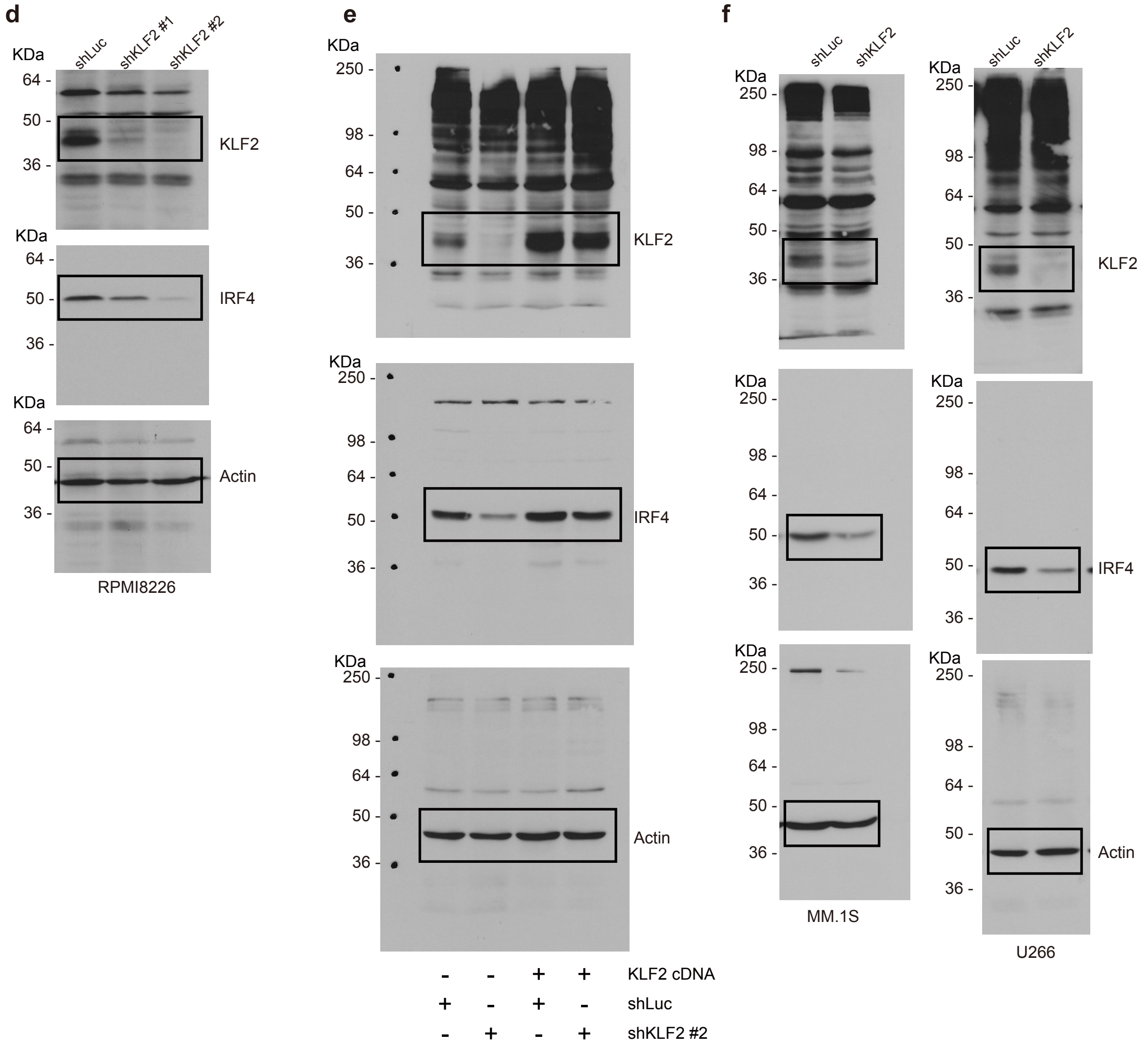

Supplementary Figure 9 | Original images of immunoblot analyses.

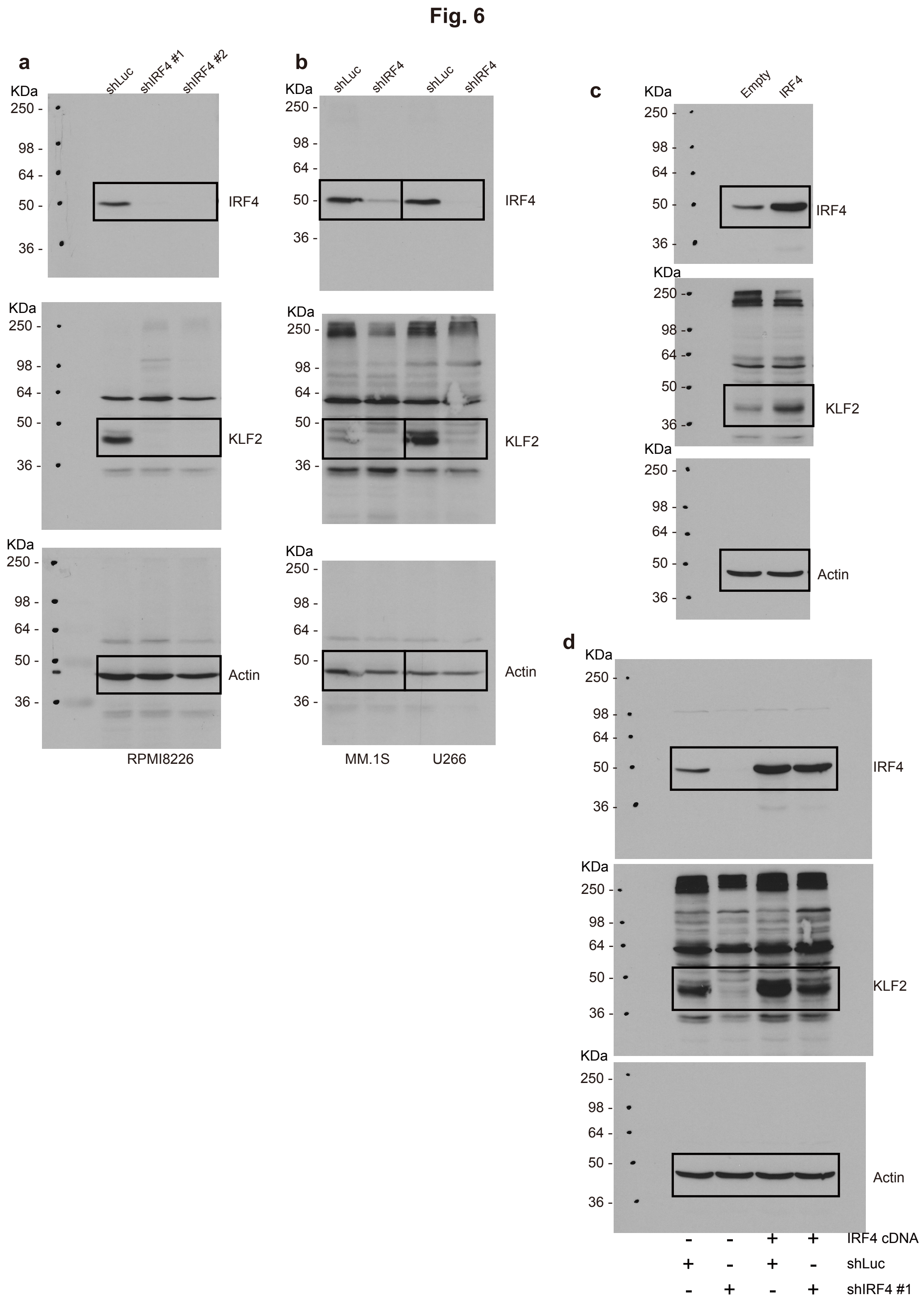

Supplementary Figure 9 | Original images of immunoblot analyses.

Fig. 7

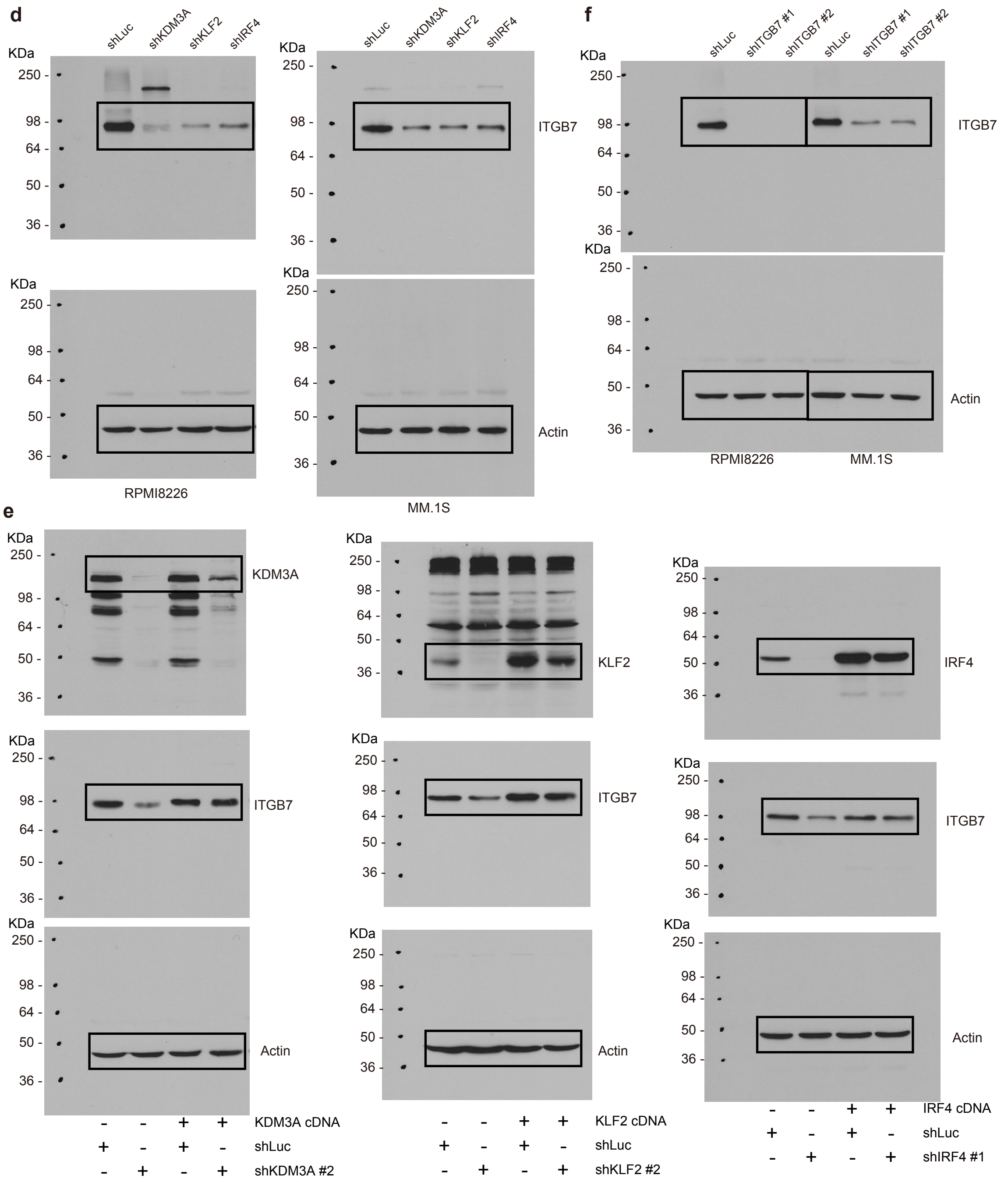

Supplementary Figure 9 | Original images of immunoblot analyses.

Supplementray Fig. 1

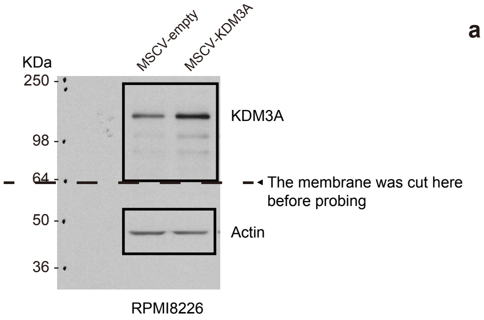

Supplementray Fig. 3

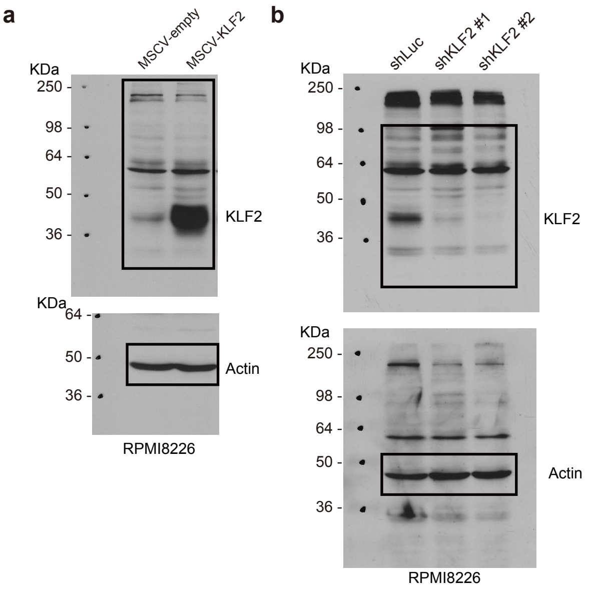

Supplementray Fig. 2d

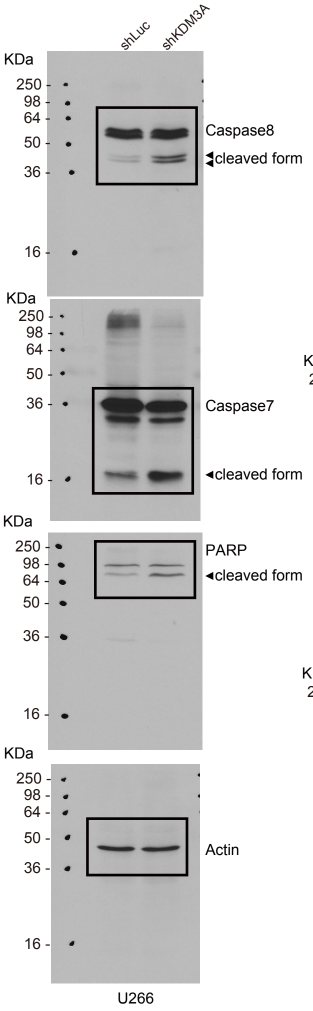

Supplementary Fig. 6b

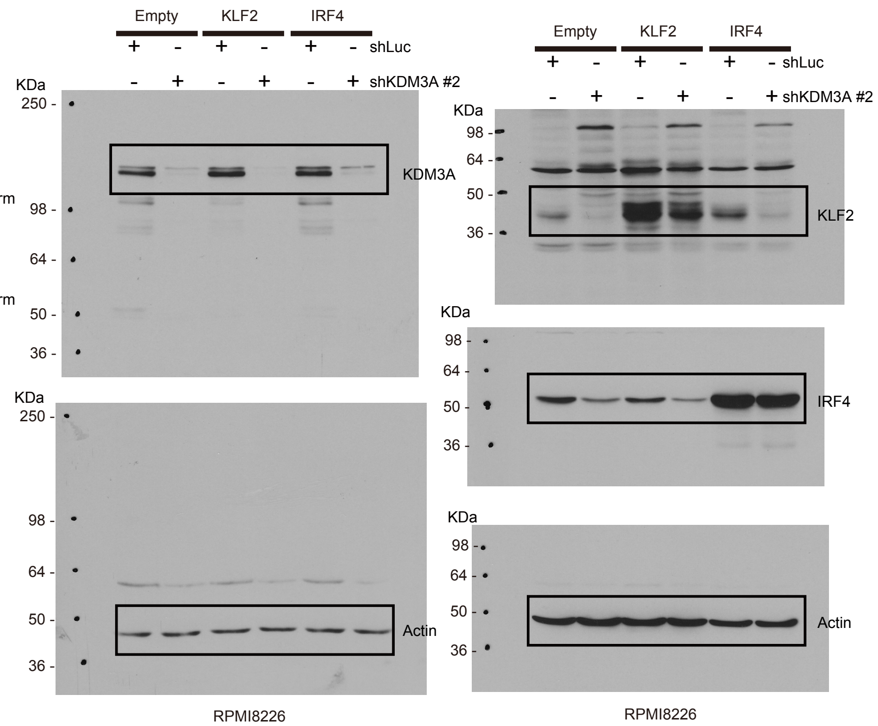

Supplementary Figure 9 | Original images of immunoblot analyses.

Supplementary Table 1. List of antibodies

**Antibodies**	**Sources**	**Catalog #**	**Applications (dilution or concentration)**
KDM3A	Novus Biologicals	NB100-77282	WB (1:4000), ChIP (8 $\mu g\ ml^{-1}$)
KLF2	Provided by Dr. Minami	N2212	WB (1 $\mu g\ ml^{-1}$), ChIP (20 $\mu g\ ml^{-1}$)
IRF4	Cell Signaling	4964	WB (1:1000)
IRF4	Santa Cruz	sc-6059	ChIP (20 $\mu g\ ml^{-1}$)
ITGB7	Sigma	HPA042277	WB (1:8000)
Actin	Santa Cruz	sc-8432	WB (1:250)
H3K9me1	Abcam	ab9045	ChIP (16 $\mu g\ ml^{-1}$)
H3K9me2	Millipore	07-441	WB (1:1000)
H3K9me2	Abcam	ab1220	ChIP (16 $\mu g\ ml^{-1}$)
H3K9me3	Millipore	07-523	WB (1:1000)
H3K9me3	Abcam	ab8898	ChIP (16 $\mu g\ ml^{-1}$)
H3	Cell Signaling	4499	WB (1:8000)
Caspase 7	Cell Signaling	9492	WB (1:1000)
Caspase 8	Cell Signaling	9746	WB (1:1000)
PARP	Cell Signaling	9542	WB (1:4000)
normal rabbit IgG	Santa Cruz	sc-2027	ChIP (control)
normal mouse IgG	Santa Cruz	sc-2025	ChIP (control)
normal goat IgG	Santa Cruz	sc-2028	ChIP (control)
rabbit IgG-HRP	Cell Signaling	7074	WB (2nd Ab) (1:2000)
mouse IgG-HRP	Cell Signaling	7076	WB (2nd Ab) (1:2000)
Apo2.7-PE	Beckmann Coulter	PN IM2088U	Flow cytometry (10 μl per sample)
IgG1(mouse)-PE	Beckmann Coulter	PN IM0670U	Flow cytometry (control)

WB; western blot, ChIP; chromatin immunoprecipitation

Supplementary Table 2. List of shRNA vectors

**Target Genes**	**Vectors**	**Clone IDs**	**Target Sequences**
KDM3A	shKDM3A #1	TRCN0000021151	GCTGGTATTTAGACCGATCAT
	shKDM3A #2	TRCN0000021152	GCTTTGATTGTGAAGCATTTA
KLF2	shKLF2 #1	TRCN0000020725	AGTTCGCATCTGAAGGCGCAT
	shKLF2 #2	TRCN0000020728	CGGCACCGACGACGACCTCAA
IRF4	shIRF4 #1	TRCN0000014763	GCCCAAATTCTCCTCTCTAAA
	shIRF4 #2	TRCN0000014764	GCCATTCCTCTATTCAAGAAT
ITGB7	shITGB7 #1	TRCN0000057718	GCACAGAGTTTGACTACCCTT
	shITGB7 #2	TRCN0000057721	GCTGAGTAAACTGATTCCTAA
Luc	shLuc (control)	TRCN0000072246	CAAATCACAGAATCGTCGTAT
RFP	shRFP (control)	TRCN0000072203	CGCGTGATGAACTTCGAGGAC

Supplementary Table 3. List of primers for cloning

**Genes**	**Primers**	**Sequences (5' to 3')**
KDM3A	Forward	GGAGCTCTTCCTGCAGGCGTGGAAACCATG
	Reverse (+ XhoI site)	CCGCTCGAGGCTGCCTGTAATTCATTTCCAATGTGC
KLF2	Forward	GGCCATGGCGCTGAGTGAACCCATCCTG
	Reverse	GTCCCGGCTACATGTGCCGTTTCATGTG
IRF4	Forward (+ EcoRI site)	GGAATTCATGAACCTGGAGGGCGGCGGCCGAG
	Reverse (+ XhoI site)	CCGCTCGAGATTGTGTCACTGCACTCCAGCCTGG
***IRF4* promoter**	**Primers**	**Sequences (5' to 3')**
IRF4 -1200	Forward (+ XhoI site)	CCGCTCGAGTAATTTTGCGATTAAGGACATCTTGG
IRF4 -520	Forward (+ XhoI site)	CCGCTCGAGGGTTCCCGGTGATGGCCTTGCCGA
IRF4 -480	Forward (+ XhoI site)	CCGCTCGAGTCCACCTCCAGTTCTCTTTGGACCA
IRF4 -160	Forward (+ XhoI site)	CCGCTCGAGCCAGCCTTCACGCCGGCCCTGAGGCT
IRF4 +14	Reverse	GAGAGTGCGAGGTGGGAAAGAGGAACT

Supplementary Table 4. List of primers for mutagenesis

**Genes**	**Primers**	**Sequences (5' to 3')**
KDM3A	Forward	TAAAGAATTTCAAGCTCTCATCGTAAAACACTTAGATGAAAGCCATC
	Reverse	GATGGCTTTCATCTAAGTGTTTTACGATGAGAGCTTGAAATTCTTTA
KLF2	Forward	CCGAGTCCGGCGGCACGGATGATGATTTGAACAGCGTGCTGGACT
	Reverse	AGTCCAGCACGCTGTTCAAATCATCATCCGTGCCGCCGGACTCGG
***IRF4* promoter**	**Primers**	**Sequences (5' to 3')**
IRF4 -181	Forward	CCAAGGGCGCGGAACCAGATCTCGGCCGCGGCAGCCCCC
	Reverse	GGGGGCTGCCGCGGCCGAGATCTGGTTCCCGCGCCCTTGG
IRF4 -496	Forward	CCCGGTGATGGCCTTGCCGAGATCTCTCCCGCAACCTCCACCTC
	Reverse	GAGGTGGAGGTTGCGGGAGAGATCTCGGCAAGGCCATCACCGGG

Underlined bases represent the specific mutations

Supplementary Table 5. List of primers for realtime PCR

**Target Genes**	**Primers**	**Sequences (5' to 3')**
KDM3A	Forward	CCGCAAGGATAAGGAGCAAC
	Reverse	CCTTCTACCCGCAACACACC
KLF2	Forward	CCAAGAGTTCGCATCTGAAGG
	Reverse	CGTGTGCTTTCGGTAGTGG
IRF4	Forward	AACAAACTGGAGAGAGACCAGACC
	Reverse	CCTCTCCAAAGCATAGAGTCACC
ITGB7	Forward	CCAGCAACGTGGTACAGCTC
	Reverse	TCAGCCTTACCCTCCCTCTTC
Cyclophilin A	Forward	TGGTTCCCAGTTTTTCATCTGC
	Reverse	CCATGGCCTCCACAATATTCA

Supplementary Table 6. List of primers for ChIP-PCR

**Target Regions**	**Primers**	**Sequences (5' to 3')**
KLF2 near TSS	Forward	GCGCTGAGTGAACCCATCCTG
(+88 bp relative to TSS)	Reverse	CCGCAGCCCACGTTCTACTAC
IRF4	Forward	GCATTTCGCACCTCGCCCTTCG
(-138 bp relative to TSS)	Reverse	CTCAGGGCCGGCGTGAAGGCT
MYOD1	Forward	GCAGTGTTCCTATTGGCCTCG
(-39 bp relative to TSS)	Reverse	GGCTTCCTCACCCCTAGCTTCT
GAPDH	Forward	CACCATTAGGGACCTTCTTGCCT
(-1.3 kb relative to TSS)	Reverse	TTCTGGGATTGCCTTTCCTGCT
KLF2 second intron 1	Forward	GGAGGTTGGGAAGAGCACTTAGA
(+1.5 kb relative to TSS)	Reverse	ACTTCCTGAGACGCAGCATCTC
KLF2 second intron 2	Forward	GAGATGCTGCGTCTCAGGAAGT
(+1.6 kb relative to TSS)	Reverse	CTGAGGGATCCTTGCCCTACAT
KLF2 -2.8 kb	Forward	GTACGTTAGTTAAGGGAGGTTGAA
(-2.8 kb relative to TSS)	Reverse	CTGAGGTACCTCTCTGCCTTCC
KLF2 -2.1 kb	Forward	GGCATCCCATATCCTTGAGGAG
(-2.1 kb relative to TSS)	Reverse	GCTATTGTGAGTTCACACAGGCTCT
KLF2 -1.6 kb	Forward	CCACATGGTCCCCAATGACTT
(-1.6 kb relative to TSS)	Reverse	GGGATTACAGGTGTGAGCCATTG
SUB1 promoter[2]	Forward	AGGCACACTGCCCAGGTTCCCTCAGTGA
	Reverse	ATCTGCAACCCTTCCTGCTTTAACAAGT

TSS; transcriptional start site

Supplementary References

1. Zhan, F. *et al.* The molecular classification of multiple myeloma. *Blood* **108**, 2020-2028 (2006).
2. Shaffer, A.L. *et al.* IRF4 addiction in multiple myeloma. *Nature* **454**, 226-231 (2008).
